# Infection-Associated Thymic Atrophy

**DOI:** 10.3389/fimmu.2021.652538

**Published:** 2021-05-25

**Authors:** Mingli Luo, Lingxin Xu, Zhengyu Qian, Xi Sun

**Affiliations:** ^1^ Department of Parasitology of Zhongshan School of Medicine, Sun Yat-sen University, Guangzhou, China; ^2^ Key Laboratory of Tropical Disease Control, Ministry of Education, Sun Yat-sen University, Guangzhou, China; ^3^ Provincial Engineering Technology Research Center for Biological Vector Control, Guangzhou, China

**Keywords:** thymus gland, atrophy, infections, immunosenescence, glucocorticoids

## Abstract

The thymus is a vital organ of the immune system that plays an essential role in thymocyte development and maturation. Thymic atrophy occurs with age (physiological thymic atrophy) or as a result of viral, bacterial, parasitic or fungal infection (pathological thymic atrophy). Thymic atrophy directly results in loss of thymocytes and/or destruction of the thymic architecture, and indirectly leads to a decrease in naïve T cells and limited T cell receptor diversity. Thus, it is important to recognize the causes and mechanisms that induce thymic atrophy. In this review, we highlight current progress in infection-associated pathogenic thymic atrophy and discuss its possible mechanisms. In addition, we discuss whether extracellular vesicles/exosomes could be potential carriers of pathogenic substances to the thymus, and potential drugs for the treatment of thymic atrophy. Having acknowledged that most current research is limited to serological aspects, we look forward to the possibility of extending future work regarding the impact of neural modulation on thymic atrophy.

## Introduction

Senility postponement is a topic of perennial importance for scientists. Aging inevitably leads to tissue damage and organ dysfunction, and individuals also become more sensitive to infections, tumors and other diseases as they age. Scientists have been studying mechanisms of human aging and searching for methods to postpone senility for many years, and several strong theories have been put forward, including the telomeric theory of aging and the free radical theory. Aging also impacts the immune system and thymic atrophy is the most obvious phenomenon of immune system degeneration.

The thymus is the main lymphoid organ of the human body. It is located anterior to the aortic arch and left brachiocephalic vein. It serves as the site for T cell maturation. The thymus parenchyma is surrounded by the thymic capsule, which extends into the parenchyma to form the lobular septum and separates the parenchyma into lobules. The thymus parenchyma consists of cortex and medulla, which are separated by the cortico-medullary junction (CMJ) (1). The cortex is constructed from thymic epithelial cells (TEC) as its framework and thymocytes filling in the interspace. The medulla is made of thymic epithelial cells, single positive (SP) thymocytes and macrophages. When common lymphoid progenitors originating from bone marrow migrate into the thymic cortex through vessels, they complete the expression of rearranged T cell receptors (TCR) in the outer cortex and change their phenotype from double negative (DN) thymocytes to double positive (DP) thymocytes; that is, from CD4-CD8- to CD4+CD8+ thymocytes. Positive selection and negative selection are two crucial processes for the continued development of DP thymocytes. Positive selection will clear DP thymocytes that cannot bind to MHC peptide, while negative selection will induce apoptosis in thymocytes that express self-reactive TCRs (2). After fulfilling the process of positive selection in the inner cortex and negative selection at the medulla, DP thymocytes become single positive thymocytes with self-tolerance; that is, CD4+CD8- or CD4-CD8+ thymocytes. SP thymocytes are then transported out of the thymus to T cell zones of the peripheral lymphoid tissues. In summary, the thymus performs these primary functions, which are essential for peripheral T cell renewal (1).

Thymic atrophy, also known as thymic involution, is inevitable upon aging, and is thus called age-associated thymic atrophy (3). In addition to human beings, thymic atrophy serves as an important characteristic aging hallmark for all species that possess a thymus. Nutrition is also involved: scientific findings indicate that zinc deficiency (4) and malnutrition (5) can cause thymic atrophy. Infection is also a vital factor. The thymus was previously thought to be an immune-privileged organ, but now it has been found that anti-pathogen responses can also be seen within thymus, causing thymic atrophy (6). Thus, infectious disease, as well as aging and nutrition, plays an important role in thymic atrophy. It is suggested that immune insufficiency is the end result of thymic atrophy (7). Thymic atrophy will lead to attenuation of immune reactions to pathogens and vaccines (3). The incident rate of tumors is positively correlated with age because of dysfunction of the monitoring of the immune system (8, 9). Typically, immunosurveillance should protect intact tissue against mutated cells that are recognized as foreign bodies (10). Besides the effects of aging, there are autoimmune diseases associated with thymic atrophy (11). Increased likelihood of pathogen-related mortality and death from opportunistic infection is also observed in patients with immune insufficiency (12). The thymus plays a crucial role in protection against COVID-19 in children (13, 14), whose thymus glands are more functional and active. Virus-specific plasma cells are accumulated in the perivascular space of the thymus (PST), located between thymic epithelial cells and blood circulation. According to the speculation of the researchers of this study (13), plasma cells, also named effector B cells, secrete IgG1 and IgG3 that are main protectors against viral infection. This may be one of the reasons that children are less susceptible to COVID-19. It is worth mentioning that there was no association between T cell development and susceptibility to COVID-19 observed in this study.

Age-associated thymic atrophy leads to decreased naive T lymphocytes, which is then followed by a series of immune sequelae mentioned above (10). This series of age-related changes that affect the immune system are defined as immunosenescence (15), whose main features are defective immune responses, increased systemic inflammation (16) and increased susceptibility to cancer (17). Age-associated thymic atrophy is characterized as expansion of the perivascular space, e.g. adipocytes and stroma. Meanwhile, the epithelial space of the thymus for thymopoiesis shrinks to less than 10% of thymic parenchyma by the age of 70 (10). Similarly, in infectious diseases, acute and chronic infection provokes alterations in inflammation and suppressive pathways that affect the function and integrity of many tissues. Depletion of thymocytes, particularly the cortical lymphocytes bearing the phenotype of double positive, is commonly observed (18). Different mechanisms have been proposed to explain age-associated thymic atrophy, including aging of the lymphocyte progenitor, defects in expression of the TCR gene and change in thymic microenvironment (10, 19, 20). However, few scientific studies have focused on the mechanism of infection related to thymic atrophy.

Here, we review the mechanism and process of thymic atrophy driven by different pathogens, including viruses, bacteria, parasites and fungi, that have been reported in recent years, with a view to elucidating research on infection-related thymic atrophy. **Tables 1**–**4** provide a summary of this work.

**Table 1:** Alterations and characteristics of virus-induced thymic atrophy.

Virus	Strain	Reference	Histological and immunophenotype alteration		Molecular characteristics
PRRSV	PRRSV-1	(21)	1. Apoptosis of CD3+ thymocytes in the cortex. 2. Increase in macrophages both in cortex and medulla.		a) Increase in TNF-α and IL-10 positive cells.
	HP-PRRSV (HuN4)	(22) (23) (24)	1. Apoptosis in bystander cells (uninfected cells) e.g. CD3+ T cells. 2. Autophagy in both infected and bystander cells. CD14+ cells and thymic epithelial cells are the main autophagic cells. 3. Depletion of DP thymocytes.		a) Decrease in IL-4. b) Increase in IL-10 and IFN-γ.
	Lena strain	(25)	1. Apoptosis in medulla and cortex. 2. Positive correlation between thymic atrophy and viral load.		a) Increase in TUNEL and cCasp3 expression in cortex. b) Increase in cCasp8 and Fas in cortex and medulla.
BVDV	non-cytopathic BVDV-1a strain 7443	(26) (27)	1. Reduction in cortex: medulla ratio 2. Increase in type I collagen deposition and vascularization.		a) Expression of receptor for BVDV, bovine CD46 in thymus.
influenza A virus (IAV)	mouse-adapted influenza A/Puerto Rico/8/34 (H1N1; PR8) strain	(28)			a) Production of IFN-γby NK cells.
	Avian Influenza A Virus	(29)			a) A consequence of digestive disorder.
	Influenza A (H1N1) pdm09	(30) (31, 32)			a) Elevation in IFN-γ secreted by activated thymic innate CD8^+^CD44^hi^ single-positive (SP) thymocytes. b) Large amounts of cytokines secreted by CD122^+^CD44^hi^Eomes^+^ innate T cells upon infected thymic DCs stimulation. c) Anti-IFN-γ therapy aids to attenuate thymic atrophy.
	Avian Influenza A Virus (AIV)-H9N2 Mixed AIV-H9N2/Influenza B Virus (IBV)	(33)	1. Congestion of medulla blood vessels and hemorrhages together with thymic atrophy.		
LCMV		(34, 35)			a) Via type I interferon and Stat2. b) IL-12 and TNF-α involved.
SIV	SIV mac251-32H	(36) (37)	1. Depletion of cortical epithelial cells and interdigitating dendrite cells.		a) Intrathymic macaques revealing phagocytosis occurs.
	Deletion of the vpu Sequences (novpuSHIVKU-1bMC33)	(38)	1. Depletion of thymocyte. 2. Destruction of thymic lobar and replaced by connective tissue.		
HIV		(39) (40)		1. Depletion of DP thymocytes and SP thymocytes. 2. Blockage of maturation process from DN4 to DP.	a) Growth hormone treatment may reverse.
Feline immunodeficiency virus (FIV)		(41) (42)		1. Depletion of CD4+ thymocytes. 2. Acute thymic atrophy in fetal cats and developed chronic thymic atrophy in neonatal cats.	
Vaccinia virus (VV)	neurotropic virulent WR strain	(43) (44)		1. Depletion of DP thymocytes.	a) 3β-hydroxysteroid dehydrogenase expressed to synthesize steroid hormones encoded by gene A44L of VV.
Murine leukemia virus (MuLV)	Friend murine leukemia virus	(45)		1. Depletion in cortex.	
	Moloney murine leukemia virus (M-MuLV)	(46)			a) Degree of thymocyte apoptosis is positive correlated with M-MuLV leukemogenesis.
	a radiation-induced strain of MuLV (RadLV-Rs)	(47)		1. At 3rd week after infection: Perivascular clusters of B-cells at the cortico-medullary junction as the first change. 2. At 4th week after infection: depletion in cortex, while a mixed population of large T- and B-cells filled the medulla.	
Chicken anemia virus (CAV)		(48) (49)		1. Depletion of DP thymocytes in cortex. 2. Apoptosis in thymic lymphoblasts.	a) Thymic cortex is the main target of CAV.
Duck enteritis virus (DEV)		(50)		1. Depletion of thymic lymphocytes. 2. Sustained immunosuppression as noted by the secondary bacterial infection.	
Mouse hepatitis virus (MHV)	Mouse hepatitis virus A59 (MHV-A59)	(51)		1. Apoptosis of DP thymocytes.	a) Thymic epithelial cells have MHV receptors. b) Infection of thymic epithelial cells led to release of cytokines.
CSFV		(52)		1. Atrophy of thymus	a) CSFV RNA was positive in the thymus when postnatal persistent infection.
MDPV		(53)		1. Atrophy of thymus.	
Newcastle disease virus (NDV)	VVNDV	(54) (55)		1. Necrosis and depletion of the lymphocytes.	a) Vitamin A dietary supplementation delayed thymic atrophy.
Marek’s disease alphaherpesvirus (MDV)	RB-1B	(56)		1. Cell death in both thymic cells replicating MDV and uninfected cells in the thymus. 2. Premature exit of DP thymocytes.	
Rabies virus		(57, 58)		1. Depletion of DP thymocytes.	a) Reversed by adrenalectomy.
Reticuloendotheliosis virus (REV)		(59)		1. Decrease in thymus index 2. Increase in thymic reticular endothelial cells, inflammatory cell infiltration and nuclear damage.	a) Increase the level of oxidative stress
Phocid herpesvirus 1 (PhHV-1)		(60)		1. Thymic atrophy of grey seals Halichoerus grypus	

PRRSV, porcine reproductive and respiratory syndrome virus; HP-PRRSV, highly pathogenic porcine reproductive and respiratory syndrome virus; BVDV, bovine viral diarrhea virus; LCMV, lymphocytic choriomeningitis virus; SIV, simian immunodeficiency virus; HIV, human immunodeficiency virus; CSFV, classical swine fever virus; MDPV, muscovy duck parvovirus; VVNDV, velogenic viscerotropic pathotype of newcastle disease virus; DP, double positive.

**Table 2:** Alterations and characteristics of bacteria-induced thymic atrophy.

Bacteria	Reference	Histological and immunophenotype alteration	Molecular characteristic
*Salmonella* Typhimurium	(61) (62) (63) (64) (65) (66)	1. Depletion of DN and DP thymocytes. DP1 and DP2 decreased significantly while the expression of Bcl2 in DP3 increased. 2. Delayed maturation of SP led to the accumulation of CD24^high^CD3^high^ SP cells. 3. Blockage of the development of DN1. 4. Apoptosis in thymic cortex in chicks, but no significant change in CD4^+^/CD8^+^ T cell ratio. 5. Thymic atrophy but maintained T cell maturation and migration in attenuated strains (SL3261) infection. Increase in naive T cells in peripheral lymphatic organs.	a) Elevation of glucocorticoid, IFN-γ and TNF-α. b) Elevation of pJNK in DP. c) Increase in IL-22, IL8L2, and CCL4 in chicks. d) Aggravation of thymic atrophy in mice lacking in receptor guanylyl cyclase C.
*Escherichia coli*	(67, 68) (69, 70) (71)	1. Decrease in thymus weight, cellularity, thymocyte viability. 2. Depletion of DP thymocytes. 3. Necrosis of thymocytes when administrated with CT or LT. 4. Apoptosis of thymocytes but increase in CD3^+^ T cells when administrated intramuscularly.	a) Increase in TNF-α, corticosteroid and LIF.
Intestinal bacteria	(72) (73)	1. Depletion of DP and SP thymocytes. 2. Inhibition of T cell egress. 3. Impaired migration of BM progenitors to thymus caused a decrease in ETPs.	a) Elevation in intrathymic S1P level. b) Decrease in serum S1P level. c) Elevation in serum IL-6 and glucocorticoids.
*Streptococcus suis*	(74)	a) Apoptosis of CD3^+^, CD14^+^ T cells, DP thymocytes and epithelial cells. b) Increase in SP cells in thymus while decrease in blood.	a) Disorder of cytokine, including IL-2, IL-6, IL-12, TNF and IL-10.
*Streptococcus pyogenes*	(75)	1. Depletion of DP thymocytes. 2. Increase in the proportion of DN and SP thymocytes. 3. Increase in Helios^+^Foxp3^+^ Treg cells and Foxp3^+^CD25^+^ Treg cells.	
*Listeria monocytogenes*	(76)	1. Apoptosis of thymocytes.	a) Increased expression of p53, Bax and c-myc genes.
*Staphylococcus*	(77)	1. Depletion of DP thymocytes. 2. Increase in expression of CD25, TCRαβ, CD3 and CD69.	
*Francisella tularensis*	(78)	1. Necrosis of DP cells in cortex.	a) Increasing TNF-α and cortisol in serum.
*Yersinia enterocolitica*	(79)	1. Depletion of DP thymocytes. 2. Increase in TCRαβ^high^ cells, especially in Vβ6^high^ and Vβ8^high^ cells.	
*Yersinia pestis*	(80)	Apoptosis of thymocytes and Jurkat T-cells.	
*Mycobacterium avium*	(81)	1. Depletion of all cell types, especially DP cells. 2. Impaired migration of BM progenitors to thymus.	a) Increase in production of nitric oxide from macrophages. b) A slight increase in corticosterone and increased sensitivity of thymocytes to glucocorticoid-induced apoptosis.
*Mycobacterium tuberculosis*	(82) (83)	1. Apoptosis in cortex and disappearance of CMJ and cortex.	a) Elevation of glucocorticoid and TNF-α. b) Increased levels of various cytokines together with disorders of the endocrine system.
*Klebsiella pneumoniae* *Pseudomonas aeruginosa* *Streptococcus pneumoniae*	(84)	1. Disappearance of CMJ. 2. Depletion of DN, DP, SP thymocytes, especially DP cells.	a) Elevation of TNF-α.

DN, double negative; DP, double positive; CT, cholera toxin; LT, heat-labile enterotoxin; LIF, leukemia inhibitory factor; SP, single positive; CMJ, cortico-medullary junction.

**Table 3:** Alterations and characteristics of parasite-induced thymic atrophy.

Parasite	Reference	Histological and immunophenotype alteration	Molecular characteristic
*Trypanosoma cruzi*	(85, 86) (87, 88)	1. Cortical thymocytes loss. 2. Apoptosis of thymocytes, especially DP thymocytes. 3. Migration disturbances. 4. Premature exit of thymocytes.	a) Decrease in IL-2. b) Increase in IL-4,5,6, IFN-γ, TNF-α.
*Leishmania infantum*	(89, 90)	1. Reduced cortical area. 2. Decreased proliferation and increased apoptosis.	a) Reduced chemotactic factors and dysregulated of migratory factors.
*Plasmodium berghei*	(91–93)	1. Loss of cortical-medullary delimitation. 2. Apoptosis of thymocytes especially DP and DN thymocytes. 3. Premature exit of thymocytes.	a) Altered thymic microenvironment with significantly increased CXCL12 and CXCR4, decreased CCL25 and CCR9. b) Increased expression of ECM component, downregulated expression of laminin and FN receptor on thymocytes surface.
*Plasmodium chabaudi*	(94)	1. Depletion of SP (CD4+ or CD8+) and δγ+ T cells.	
*Plasmodium yoelii lethal* (17XL)	(95)	1. Depletion of DP and CD4+ SP thymocytes. 2. Reduced proliferation of DP thymocytes. 3. Downregulation of CD8 expression.	
*Plasmodium yoelii nonlethal* (17XNL)	(95)	1. Depletion of DN, DP, and CD4+ SP thymocytes. 2. Reduced proliferation of DN and DP thymocytes.	
*Angiostrongylus cantonensis*	(96, 97)	1. Depletion of CD3+, CD4+, and CD8+ T cells. 2. Increased thymocytes and thymic epithelial cells apoptosis.	
*Schistosoma mansoni*	(98)	1. Cortical thymocytes loss. 2. Loss of distinction in the corticomedullary region.	
*Schistosoma japonicum*	(99, 100)	1. Thymic atrophy but with both CD8+CD28- T cells and CD4+CD28- T cells increased.	a) Thymocytotoxic autoantibodies was found in thymus.

DP, double positive; ECM, extra-cellular matrix; SP, single positive; DN, double negative.

**Table 4:** Alterations and characteristics of fungus-induced thymic atrophy.

Fungus	Reference	Histological and immunophenotype alteration	Molecular characteristic	
*Paracoccidioides brasiliensis*	(101) (102) (103)	1. Structural disorganization and intense inflammatory infiltration. 2. Enhanced migratory ability of thymocytes. 3. Depletion of all subpopulations. 4. Disorder of selection process. 5. Formation of granuloma in chronic infection.	a) Invasion into thymus by yeast. b) Increase in IL-2, IL-7, IL-17, TNF-α and AIRE. c) Higher level of inflammatory cytokines, inflammasome activity and gene expression of caspase-1 and caspase-8. d) Mature T cells re-entered infected thymus.
*Aspergillus fumigatus*	(104)	Apoptosis of thymocytes.	
*Fusarium*	(105)	Depletion of each subgroup decreased.	
*Saprolegnia*	(106)	Atrophy of fish thymus.	
*Candida albicans*	(107)	Apoptosis of thymocytes.	C. Albicans FLO8-deficient (*flo8*) mutant enhances the production of IL-10 by dendritic cells and macrophage to attenuate apoptosis of T cells.

## Viruses

Viruses can induce thymic atrophy through different mechanisms. In general, direct and indirect cell death induced by infection are two common means of thymic atrophy. The indirect cell death pathway includes cytokine alteration and glucocorticoid release. Here we present some typical examples, with further examples summarized in **Table 1**.

### Porcine Reproductive and Respiratory Syndrome Virus (PRRSV)

Porcine reproductive and respiratory syndrome (PRRS) is characterized as reproductive failure and respiratory disorder in sows, as well as immune senescence (108). Porcine reproductive and respiratory syndrome virus (PRRSV) is the pathogen of PRRS, which can be divided into two types: PRRSV-1 (formerly European type) and PRRSV-2 (formerly North American type) (21). PRRSV-1 has been further divided into four different subtypes according to sequence analysis (109) based on highly diverse ORF5 and ORF7 genetic sequences of PRRSV-1.

Highly pathogenic (HP) PRRSV strains have been identified in both PRRSV-1 and PRRSV-2 so far (110). Piglets infected in utero with the SD 23983 PRRSV strain demonstrated severe thymic atrophy, which leads to immunosuppression (111). HP-PRRSV (HuN4 strain) has a much stronger tropism in the thymus than low pathogenic PRRSV (112). Researchers have demonstrated that HP-PRRSV (HuN4 strain) can lead to thymic atrophy, and can cause a significant loss of DP thymocytes when compared to low pathogenic PRRSV (CH-1a strain) (22). HP-PRRSV attenuated strain shows a reduced ability to induce thymocyte atrophy (113), indicating that pathogenicity is positively correlated with the degree of thymic atrophy. Similarly, as for low virulent PR11 and high virulent PR40 PRRSV1 subtype 1 strains, thymic atrophy is more intense in the PR40 group (114). However, CD3^+^ T cells, the main apoptotic component, are not infected with HP-PRRSV, and viral nucleotides are only found in CD14^+^ monocyte/macrophages (108), the main replicating sites of PRRSV in the thymus (21). This analysis suggests an indirect route to apoptosis induction, which may be correlated with cytokine release (108). Similar results in PRRSV-1 support the hypothesis above, especially in PRRSV-1 SUI-bel strain. Apoptosis of CD3^+^ thymocytes in the cortex was noted and the number of TNF-α and IL-10 immuno-stained cells showed an increase in the infected group (21). There was also an increase in the number of macrophages in both the cortex and the medulla of the thymus (21). Cytokines released by macrophages may play an important role in thymocyte apoptosis.

Further research suggests that both apoptosis and autophagy occur in the thymus of piglets infected with HP-PRRSV (23). The main autophagic components are thymic epithelial cells, along with CD14^+^ cells (23). HP-PRRSV only induces apoptosis in bystander cells (uninfected cells), while autophagy occurs in both infected and bystander cells (23).

A recent hypothesis suggests that thymic antigen presenting cells (TAPCs) infected by PRRSV interact with DP thymocytes, which results in an acute deletion of DP thymocytes and a poor ability to recognize PRRSV and other pathogens (115).

### Influenza A Virus

Influenza A virus (IAV) is one of the most common and important viruses that induces recurrent epidemics and causes high morbidity and mortality in the population. IAV infection can also induce thymic atrophy. Research involving mouse-adapted influenza A demonstrated that NK-cells affect thymic atrophy *via* IFN-γ production (28). The expression of proinflammatory cytokines is elevated in influenza A (H1N1) pdm09-infected thymus tissue (30), the most significant of which is IFN-γ secreted by activated thymic innate CD8^+^CD44^hi^ SP thymocytes (30). IAV can be presented by thymic DCs (30), resulting in rapid secretion of large amounts of cytokine by CD122^+^CD44^hi^Eomes^+^ innate T cells (31). This mechanism may also participate in IAV-induced thymic atrophy.

Avian IAV can lead to thymic atrophy and severe diarrhea in chicks, but thymic atrophy in such cases is thought to be the result of a digestive disorder (29).

### Immunodeficiency Virus

Immunodeficiency virus is a retrovirus that affects a variety of animals in addition to humans (47). In human acquired immunodeficiency syndrome (AIDS), severe thymocyte depletion restricts T cell production in the thymus, which is the main pathogenesis of human immunodeficiency virus (HIV). Mice transgenic for HIV-1 Tat protein show an acute thymic atrophy, which is characterized by depletion of cortex and loss of the cortico-medullary border. DP thymocytes and SP thymocytes are significantly reduced as a result of the blocked maturation process from 4^th^ stage DN to DP (39).

In other animal models, Simian immunodeficiency virus (SIV)-induced thymic atrophy is characterized by severe depletion of cortical epithelial cells and interdigitating dendrite cells (36). SIV-induced thymic atrophy exhibits shrinkage of the thymic capsule, as opposed to age-related thymic atrophy that is characterized by an unaltered thymus capsule filled with adipose tissue (36). The Vpu protein of HIV-1 enhances the release of virion from infected cells. However, the Simian–Human Immunodeficiency Virus with the deletion of Vpu sequence (novpuSHIV_KU-1bMC33_) can still result in severe thymic atrophy in macaques (38). In macaques infected with novpuSHIV_KU-1bMC33,_ depletion of thymocytes and destruction of thymic lobes was observed. Lobes were replaced by connective tissue (38), and further research is needed to fully understand the mechanism. HIV can be vertically transmitted to the subsequent generation through the parental pathway. In cats infected with feline immunodeficiency virus and serving as animal models for lentiviral immunodeficiency disease (41), fetal cats show acute thymic atrophy at birth with peak viremia while neonatal cats develop chronic thymic atrophy with low-level productive infection and viremia (42). Interestingly, thymic atrophy in fetal cats is rapid and transient, rebounding 46 days after infection (42). In humans, a reduction in thymus size has been observed in children who have been exposed to HIV but do not have qualitative immunodeficiency (116). Recent findings have showed that treatment of HIV-1 infected adults with growth hormone might reverse thymic atrophy (40).

### Other Viruses

Many other domestic viruses are also reported to cause thymic atrophy. The thymus is one of the target organs of viruses. Apoptosis is induced by infection, and alteration in the thymic microenvironment also contributes to thymic atrophy.

Marek’s disease is a lymphoproliferative disease of chickens, whose pathogen is Marek’s disease alpha herpes virus (MDV) (117). Thymic cells infected with replicating MDV subsequently show significant apoptosis. MDV infection also promotes cell death of uninfected cells in the thymus. However, in cases of MDV infection, the number of T-cells in the blood is not significantly decreased, on the contrary, the lymphocytosis of CD4^+^ and CD8^+^ T cells occurs (56). At present, thymic atrophy is a key indicator to evaluate a new Marek’s disease vaccine (117–119).

Chicken anemia virus (CAV) can cause anemia in chickens, and thymic atrophy occurs in the CAV infected chicks (120). Depletion of lymphocytes in the thymus cortex is observed, which is characterized by a loss of CD4^+^CD8^+^ DP thymocytes (48). CAV antigens, VP1 and VP3, are detected positively in thymic cortex, the main target of CAV (49). In cases of CAV infection, caspase 3 is detected mainly in cells that are compatible morphologically with thymic lymphoblasts (49) indicating that thymic apoptosis mainly occurs in lymphoblasts.

In mouse hepatitis virus (MHV) infection, the MHV receptor (MHVR) glycoprotein is detected on thymic epithelial cells but not on T lymphocytes. Infection of thymic epithelial cells leads to release of cytokines, which results in alteration of the microenvironment and apoptosis of immature CD4^+^CD8^+^ DP lymphocytes (51).

In one study, calves were pre-infected with Bovine viral diarrhea virus (BVDV) and secondly infected with bovine herpesvirus 1 (BHV-1). BVDV pre-infected calves showed reduction in cortex: medulla ratio, with severe cortical thymic atrophy, increased type I collagen deposition and increased vascularization (26). The cellular receptor for BVDV, bovine CD46, can be purified from the thymus (27), which suggests that thymic cells may be directly infected by BVDV through the CD46 pathway.

Other mechanisms are also implicated in virus related thymic atrophy. Infection with Vaccinia virus (VV) induces severe thymic atrophy characterized by reduction of CD4^+^CD8^+^ DP thymocytes in the thymus (43), and gene A44L of VV is reported to be responsible for encoding 3β-hydroxysteroid dehydrogenase which can synthesize steroid hormones. Thus, VV may affect thymic atrophy by regulating the production of steroid hormones itself (44). Lymphocytic choriomeningitis virus (LCMV) is reported to induce thymic atrophy *via* type I interferon and signal transducer and activator of transcription 2 (Stat2) (34). Induction of TNF together with high concentration of IL-12 seems to explain the immunotoxicities induced by LCMV infection (35). What’s more, during LCMV infection, self-reactive T cells are able to leave the thymus and enter the peripheral circulation due to impaired negative selection (34). Classical swine fever virus (CSFV) in pigs (52), Muscovy duck parvovirus (MDPV) in ducks (53) and Newcastle disease virus (NDV) in cockerels (54) are all reported as causing thymic atrophy. However, the exact mechanism in each of these cases is still being studied.

In brief, PRRSV is a well-studied virus in the field of virus-related thymic atrophy. Its pathogenesis involves replication of virus in the thymus and changes in the thymus microenvironment and cytokines, which induces apoptosis or autophagy. While the mechanisms of thymic atrophy induced by other viruses have not yet been fully studied, some studies only confirmed that certain viruses may cause thymic atrophy, while others focused on the pathological changes of the thymus after infection. Additionally, some studies focused on changes in thymocyte subpopulations while others focused on changes in the thymus microenvironment. Thus, more comprehensive studies are needed to reveal the mechanisms by which other viruses induce thymic atrophy. **Table 1** shows the specific characteristics of thymic atrophy caused by different viruses.

## Bacteria

Infections with bacterial pathogens are also known to result in thymic atrophy. Thymic atrophy induced by bacterial infection involves a variety of mechanisms, and the exact mechanism will depend on the type of bacteria that cause the infection. Therefore, it is crucial to determine the specific pathway and specific results of thymic atrophy caused by a given bacteria for the study and prevention of thymic atrophy induced by the bacteria.

### Salmonella Typhimurium

The mechanism of thymic atrophy caused by *Salmonella Typhimurium* (*S. Typhimurium*) has been well studied. During *S. Typhimurium* infection in mice, there is a significant decrease in the number of DP thymocytes, but no obvious change in the number of SP thymocytes and peripheral lymphocytes (61). Majumdar et al. (62) further studied the changes in different subsets of thymocytes during *S. Typhimurium* infection, which revealed that subsets of DP1 (CD5^lo^CD3^lo^) and DP2 (CD5^hi^CD3^int^) were mainly reduced, while the surviving DP3 thymocytes (CD5^int^CD3^hi^) expressed elevated amounts of intracellular Bcl2. Corresponding to that, SP thymocytes were more resistant to depletion but their maturation was delayed, leading to accumulation of CD24^hi^CD3^hi^ SP thymocytes. A blockade in the developmental pathway of DN thymocytes was also observed and all the phenomena above were enhanced by IFN-γ. Indeed, *S. Typhimurium* indirectly leads to depletion of DP thymocytes by increasing endogenous glucocorticoid and IFN-γ. This induces apoptosis *via* various mechanisms involving mitochondrial membrane depolarization and activation of caspase-3, but not Fas/FasL pathway (61). Subsequent studies showed that the glucocorticoid and IFN-γ mediated pathways led to the downstream activation of c-Jun NH2-terminal kinase (JNK) in DP thymocytes, which was crucial for apoptosis of immature thymocytes during infection. In turn, there are some feedback regulations of JNK that elevate the serum level of glucocorticoid and IFN-γ (63). Other studies showed that lipopolysaccharides (LPS) could be the main pathogenic factor of thymic atrophy in chicks during infection with *S. Typhimurium*, and that the process might involve TLR4-FOS/JUN signaling pathway (64). Interestingly, when attenuated strains were used to induce thymic atrophy, different results emerged. Ross et al. (65) found that thymic atrophy induced by attenuated strains of *S. Typhimurium* was neither dependent on regulation of endogenous glucocorticoids nor IFN-γ. Furthermore, the maturation and output of thymic T cells were maintained during atrophy. Once bacterial numbers decreased, thymocyte numbers recovered, indicating that the thymus has some adaptability to attenuated strains and can maintain its function during infection.

### *Escherichia coli* and Gastrointestinal Microbiota

Thymus weight, cellularity, thymocyte viability and absolute number of DP thymocytes decrease after injection of *Escherichia coli* (*E. coli)* or *E. coli*-derived LPS into mice (67, 68). TNF-α and corticosterone play an important role in the apoptosis of thymocytes *in vivo* induced by LPS of *E. coli* (68). Other studies found that the LPS-induced thymic atrophy during *E. coli* infection was significantly mediated by leukemia inhibitory factor (LIF, a member of the IL-6 cytokine family) which was able to enhance systemic and intrathymic corticosteroids (67). When cultured with T cells *in vitro*, high doses of the cholera toxin (CT) or its B sub-units (CT-B) from *E. coli* induce apoptosis by increasing cAMP or by cross-linking of cell surface GM1 and CT-B (121, 122). Intravenous administration of CT or heat-labile enterotoxin (LT) induce decreased thymus weight due to *in vivo* necrosis of thymocytes, but not apoptosis (69, 70). However, intramuscular administration of LT to mice induces apoptosis in the thymus, which may be the result of the body’s immune response, rather than the toxin directly interacting with thymocytes through circulation (71).

Thymic atrophy is observed in a model of sepsis induced by cecal ligation and puncture (CLP) in mice. Apoptotic thymocytes increase sphingosine-1-phosphate (S1P) in the thymus, which disrupts the normal S1P gradient *in vivo*, leading to thymic involution and inhibition of T cell emigration. S1P also increases IL-6 and aggravates the apoptosis of thymocytes (72). In another sepsis model induced by CLP or by continuous injection of *E. coli*-derived LPS, Kong et al. (73) observed some phenomena from the perspective of early T lineage progenitors (ETPs). In this model, there was a depletion of ETPs, which was due to decreased expression of some chemokine receptors such as CCR7, CCR9 and P-selectin glycoprotein ligand-1 on the surface of bone marrow (BM) progenitors for ETPs, contributing to impaired homing capacity of these cells. There was also a defect in lymphoid lineage commitment. Progenitors were more likely to differentiate towards the myeloid rather than the lymphoid lineage.

### Streptococcus

Wang et al. (74) found that *Streptococcus suis* (*S. suis*) induced thymus apoptosis by promoting apoptosis of CD3^+^, CD14^+^, DP and thymic epithelial cells. By destroying mitochondrial function and releasing cytochrome C (CytC) and apoptosis-inducing factors (AIF) into the cytosol, *S. suis* triggers p53-dependent apoptosis. Caspase-dependent pathway is another way to induce thymocyte apoptosis during *S. suis* infection. The bioavailability of L-arginine is an important condition for thymogenesis (123). Studies have shown that L-arginine is an important regulator of the mTOR signaling pathway (124), which is essential for the development of T cells (125). Based on this theory, a Russian team found that *Streptococcus pyogenes* infection could induce thymic atrophy in mice and confirmed that this atrophy was inextricably associated with the reduction of L-arginine in the bloodstream by arginine deiminase produced by *Streptococcus pyogenes* (75). In fact, many pathogens that cause thymic atrophy can express products that reduce L-arginine in the blood (126).

### Staphylococcus

Lin et al. (77) reported that staphylococcal enterotoxin B (SEB) could induce thymocyte apoptosis, and I-E molecule on antigen-presenting cells played an important role in this process. According to this report, in addition to a decrease in the number of DP thymocytes, there was a higher expression of surface markers such as CD25, TCRαβ, CD3, and CD69 in thymocytes after SEB injection, which was not observed in I-E negative mice. This report also mentions that staphylococcal enterotoxin A (SEA) can also induce thymocyte apoptosis independent of I-E expression.

### Mycobacterium

In the study of mice infected with *Mycobacterium avium* (*M. avium*), it was found that the synergistic effect of nitric oxide produced by IFN-γ-activated macrophages and glucocorticoid played a major role in inducing thymic atrophy (81). During *M. avium* infection, the number of thymocytes decrease, including earliest thymic precursors. This suggests defects in the homing of BM progenitor cells and their differentiation into thymocytes (81). In theory, *Mycobacterium tuberculosis* (*M. tuberculosis*) can also induce thymic atrophy. *M. tuberculosis* not only elevates levels of IFN-γ, IL-6 and glucocorticoids, but also disrupts the endocrine system, increasing growth hormone, thyroid hormone, estradiol and prolactin (82). Abnormal elevations of these hormones may indicate impaired thymus function (127–131). Injections of *M. tuberculosis*-derived cord factor (CF) and LPS instead of sulfides were proved to induce thymic atrophy (83). TNF-α and corticosterone increased remarkably after LPS injection, but they did not change significantly after CF injection. However, administration of anti-TNF-α antibody almost completely inhibited thymic atrophy caused by CF (83). Additionally, anti-TNF-α antibodies can also completely or partially inhibit thymocyte apoptosis caused by various gram-negative and gram-positive bacteria, such as *E.coli*, *Klebsiella pneumoniae*, *Pseudomonas aeruginosa* and *Streptococcus pneumoniae* (84). In this regard, the TNF-α signal pathway seems to be one of the common pathways leading to thymocyte apoptosis in various bacteria.

### Other Bacteria

Merrick et al. (132) reported that after *Listeria monocytogenes* (LM) infection in mice, significant lymphocyte apoptosis was found in the T cell zones of the lymph nodes and spleen. Subsequently, Chen et al. (76) reported that P53, Bax and c-myc genes might co-regulate LM-induced thymocyte apoptosis. The relevant mechanisms need to be further studied.

Low dose aerosol infection with type A *Francisella tularensis* (*F. tularensis*) induces thymic atrophy in mice, where *F. tularensis* can be found in thymic tissue with large numbers of DP cells undergoing necrosis instead of apoptosis. This process is under the regulation of corticosteroids and TNF, rather than Fas (78). It has also been reported that, in the early stage, intraperitoneal injection of high-dose *F. tularensis* caused thymic atrophy through a series of stress responses, but no bacteria were found in the thymic tissue (133).

Intraperitoneal administration of culture supernates from *Yersinia enterocolitica* (*Y. enterocolitica*) causes thymocyte apoptosis and increases the proportion of TCRαβ^high^ cells in mice, which is related to the increase in the percentage of cells with high levels of Vβ6 and Vβ8 TCRs (79). In addition, LPS is not involved in thymic atrophy induced by *Y. enterocolitica* (79). The V antigen of *Yersinia pestis* can interact with receptor-bound human IFN-γ located in thymocytes, triggering thymocyte apoptosis *in vitro* (80). Again, the specific mechanism here remains to be clarified.

In general, studies on bacteria-induced thymic atrophy are relatively complete, and some of them even delve into the level of signaling pathways and gene expression. Considering that different bacteria have different invasiveness and produce different toxins, the mechanisms of thymic atrophy induced by different bacteria cannot be simply classified. In other words, the mechanisms by which one type of bacteria induces thymic atrophy are often different from the mechanisms utilized by other bacteria. Therefore, it is important to explore the specific mechanisms of thymic atrophy caused by each type of bacteria, which puts forward new requirements for the future research of thymic atrophy. **Table 2** shows the specific characteristics of thymic atrophy caused by different bacteria.

## Parasites

Parasite-induced thymic atrophy is associated with *Trypanosoma cruzi, Leishmania infantum, Plasmodium, Angiostrongylus cantonensis*, and *Schistosoma*. This kind of thymic atrophy is related to apoptosis of thymocytes (especially DP thymocytes), reduced proliferation of thymocytes, premature exiting of thymocytes and alteration of the thymic microenvironment, which may be the direct effect of parasites. Parasites can interrupt hormone balance to induce thymic atrophy indirectly. More detailed mechanisms of some parasite-induced thymic atrophy are yet to be determined.

### Trypanosoma cruzi

The hemoflagellate protozoan *T. cruzi* causes Chagas disease, a potentially life-threatening disease with complications such as cardiomegaly, gastrointestinal disease, and peripheral neuropathy in some cases (134). *T. cruzi* is transmitted by the vector of blood-sucking insects of the subfamily *Triatominae* and usually found in South America, Mexico, the United States, Europe and the Western Pacific region (135, 136). It attacks human organs like the thymus, spleen and lymph nodes.

Several studies have revealed thymic atrophy during *T. cruzi* infection. Once infected by *T.* cruzi, a distinct thymic atrophy occurred with loss of cortical thymocytes, apoptosis of thymocytes (especially DP thymocytes) (137), migration disturbances, premature exiting of thymocytes, decreased IL-2 level and increased IL-4,5,6, IFN-γ, TNF-α level (85–87). The mechanism underlying premature release of DP thymocytes is being studied. Initially it was believed that it was related to negative selection, but recent research has confirmed that the negative selection is normal during experimental *T. cruzi* infection (138). Experiments revealed that the key intrathymic factors essential for promoting negative selection of thymocytes, such as the expression of autoimmune regulator factor (AIRE) gene, tissue-restricted antigen (TRA) gene and proapoptotic Bim protein, remain normal during acute Chagasic thymic atrophy (138). Subsequent experiments further confirmed this by injecting ovalbumin (OVA) peptide into OVA-specific TCR transgenic mice with or without infection-induced thymic atrophy, in which infection promoted apoptosis of OVA-specific thymocytes (138). Except for DP thymocytes, researchers found an increase in peripheral CD4-CD8- (DN) thymocytes in patients with severe cardiomyopathy of Chagas disease. In the thymus of *T. cruzi* infected mice, researchers detected decreased transcription of S1P kinase 1 and 2 genes associated with S1P biosynthesis and increased transcription of the S1P lyase gene associated with S1P inactivation, indicating decreased intrathymic levels of S1P. However, DN thymocytes upregulate S1P receptor during infection, resulting in DN thymocytes migrating to peripheral blood where the level of S1P is relatively higher (139, 140). The trans-sialidase of *T. cruzi* can activate the MAPK-JNK pathway and actin filament mobilization in thymocytes, modulating the adhesion of thymocytes to TECs and their migration towards extracellular matrix, or modulating the escape of immature thymocytes during infection (141). Abnormal migration and export of thymocytes are not only related to the cell itself, but also to the surrounding microenvironment. Scientists found that the TECs infected by *T. cruzi* exhibited an increased expression of extra-cellular matrix (ECM), especially the fibronectin (FN), which led to deposits of ECM and enhancement of TECs-infection and thymocyte-TECs interaction. This may be related to the abnormal migration and export of abnormal thymocytes (142). Pe´rez et al. (85) unraveled that TNF-α together with FN triggered alterations in thymocyte migration and promoted the export of premature thymocytes, which linked the increased TNF-α (induced by *T. cruzi* infection) and fibronectin deposits with abnormal migration and export of thymocytes. Galectin-3, produced mainly by TECs, is also found to accelerate the premature death of thymocytes (143).

In addition to SP thymocytes, the thymus is also the site of differentiation of natural Tregs. *T. cruzi* infection gives rise to a noticeable loss of Tregs and results in localization, phenotypic, and functional abnormalities of the remaining Tregs (144). In addition, the differentiation of CD4+Foxp3+ T cells in peripheral blood is impaired due to an abnormal Th1-like phenotype and functional changes caused by *T. cruzi* infection, which may aggravate the immune imbalance and further affect the progression of the disease (144, 145).

Researchers further found that *T. cruzi* infection could induce thymic atrophy through affecting hormones. During infection, cytokines such as IL-1, IL-6, and TNF-α are obviously elevated, which stimulates the hypothalamic-pituitary-adrenal (HPA) axis and then increases the production of glucocorticoid, which is the main course of thymic atrophy (87, 146). For instance, elevation of IL-6 is found to be involved in the disruption of the differentiation of DN (88). Increased glucocorticoid can contribute to DP thymocyte apoptosis *via* caspase-8 and caspase-9 (147) and mediate premature release of DP thymocytes to the periphery, which may lead to autoimmune responses such as myocarditis (148). These effects can be reversed largely by blocking glucocorticoid receptors through steroid receptor antagonist RU486, resulting in an increase in thymus weight and the proportion and number of DP thymocytes. However, these effects cannot be reversed in TNF receptor 1 and 2 knockout mice (87, 147, 149). Eduardo Roggero et al. (150) considered that endogenous glucocorticoid was protective for the host during infection, given the fact that blockade of glucocorticoid receptor by RU486 resulted in elevated TNF-α level and accelerated death in experimental mice.

As noted, glucocorticoids induce apoptosis of thymocytes. In contrast, prolactin, another stress-induced hormone, antagonizes the apoptosis mediated by glucocorticoid (151–153). Glucocorticoid and prolactin have a mutual influence on thymic atrophy during *T. cruzi* infection. Researchers confirm that systematic prolactin impairment during infection increases the level of glucocorticoid, leading to thymic atrophy. Injection of metoclopramide (MET) enhances prolactin secretion, limiting the glucocorticoid-induced thymic atrophy and the export of immature DP thymocytes to the periphery (154).

Sex steroids also play a significant role in thymic atrophy during *T. cruzi* infection. Testosterone is an immunosuppressor, while dehydroepiandrosterone (DHEA) is an immunopotentiator that counteracts immunosuppressive effects of glucocorticoid (155, 156). Testosterone promotes the synthesis and release of thymogenic glucocorticoids by upregulating enzymes involved in glucocorticoid synthesis, leading to anti-proliferative and apoptotic responses of thymocytes (157). Another study showed that testosterone was involved in the apoptosis of thymocytes induced by exogenous changes of the surface sialylation, which is catalyzed by trans-sialidase (virulence factor of *T. cruzi*) *in vivo*, where the alternated sialyl residue interacts with TECs. This apoptosis process involves caspase-3 (158). Treatment with DHEA has been reported to enhance thymocyte proliferation and reduce TNF-α production during the acute phase of infection (159). Interestingly, in another study, DHEA treatment induced thymic atrophy *via* classical estrogen signaling pathways (160). To date, the mechanism of action of DHEA is unclear and more research is needed to elucidate its role.

To better understand the mechanism of thymic atrophy caused by *T. cruzi* infection, researchers tried to understand it by using molecular patterns. They found that 29 microRNAs in infected TEC were expressed differently in genes related to cell death, chemotaxis and adhesion. This highlighted that microRNAs may be mediators of thymic atrophy during *T. cruzi* infection (161). For example, the elevated level of miR-10a in TEC, which participates in T cell differentiation in the thymus through TGF-β stimulation, may influence thymic atrophy during infection (161, 162).

### Leishmania infantum

Leishmania infection causes leishmaniasis, which is transmitted *via* female phlebotomine sandflies. Zoonotic leishmaniasis shows different clinical forms such as visceral leishmaniasis, cutaneous leishmaniasis, or muco-cutaneous leishmaniasis (163). *L. infantum* is the main zoonotic pathogen that infects humans, usually in the form of visceral leshmaniasis (163).

Thymic atrophy is also observed in *L. infantum* infected malnourished mice. As a precondition, malnutrition has a deleterious effect on the T cell-mediated immune response to *Leishmania infantum* infection. *L. infantum* infection combined with protein malnutrition leads to decreased chemotactic factors, dysregulation of migratory factors, decreased cortical area, decreased proliferation and increased apoptosis. Altered protein abundance suggests that a dysfunctional thymic microenvironment contributes to thymic atrophy (89, 90).

### Plasmodium

Plasmodium is the cause of malaria and is transmitted through the bite of anopheles mosquitoes (164). Malaria has symptoms like fever, chills, headache, body-aches, cough, diarrhea and, in severe cases, brain, lung or kidney damage (165). *Plasmodium berghei* and *Plasmodium chabaudi* are rodent malaria parasites, widely used in mice models of malaria infection (166).

*P. berghei* is often observed in the thymus of infected experimental mice (91). *P. berghei* infection results in pronounced loss of thymus weight, loss of cortical-medullary delimitation, apoptosis and premature egress of thymocytes, especially DP and DN thymocytes (91, 92). Based on the degree of apoptosis, the apoptosis pattern can be divided into three types: starry-sky pattern of diffuse apoptosis maintaining cortical-medullary structure, intense apoptosis with cortical atrophy and severe cortical thymocyte depletion resulting in cortical-medullary inversion (167). During *P. berghei* infection, thymic atrophy is accompanied by changes in the microenvironment, where CXCL12 and CXCR4 are significantly increased, CCL25 and CCR9 are significantly decreased, the expression of the ECM component is increased, and the expression of laminin and FN receptor on thymocyte surfaces are downregulated, thereby altering the migration pattern of thymocytes (93).

Similarly, *Plasmodium chabaudi* causes thymic atrophy, and the number of mature, SP (CD4+ or CD8+) and δγ+ T cells in the thymus decreases (94). Recently, the effect of *Plasmodium yoelii* lethal (17XL) and *Plasmodium yoelii* nonlethal (17XNL) on the thymus has been illuminated. During *P. yoelii* lethal (17XL) infection, the decrease of DP and CD4+ SP thymocytes, the reduced proliferation of DP thymocytes and the downregulation of CD8 expression on the thymic T-cell subpopulations were observed. However, during *P. yoelii* nonlethal (17XNL) infection, an earlier decrease of DN, DP, and CD4+ SP thymocytes and the reduced proliferation of DN and DP thymocytes were observed. This effect is reversible, but it is even greater when parasitemia is much lower. In addition, in Plasmodium infection, TNF-α has a protective effect on the thymus, while the increase of TNF-α during *T. cruzi* infection exacerbates thymic atrophy (95). Interestingly, the co-infection of malaria and cutaneous leishmaniasis may influence the outcome of thymic atrophy. Researchers found that thymus glands co-infected with *Leishmania braziliensis* and *P. yoelii* recovered earlier from atrophy than those infected with *P. yoelii* alone (168).

### Angiostrongylus cantonensis

*Angiostrongylus cantonensis*, a rat lungworm, causes human angiostronglyliasis. Humans, its non-permissive hosts, are infected by ingesting undercooked snails or slugs, paratenic hosts such as prawns, or infected larvae-contaminated vegetables (169). It usually induces eosinophilic meningitis in humans (170). Recent research found that it could also cause thymic atrophy in the non-permissive host, which may be explained by two mechanisms. One is the activation of the HPA axis, which impairs thymocyte development (96). Another is the direct action of soluble antigen from *A. cantonensis*, which leads to increased apoptosis of thymic stromal cells and thymocytes (97).

### Schistosoma

Schistosoma is mainly prevalent in endemic regions of Africa and is also seen in the Middle East, Caribbean, South America, and South East Asia (171). The most common pathogenic species include *Schistosoma haematobium*, *Schistosoma mansoni* and *Schistosoma japonicum*, which cause urinary, intestinal and hepatosplenic schistosomiasis respectively (172, 173). Most infected people show no, or limited, atypical symptoms such as abdominal pain, diarrhea, anorexia, ascites, splenomegaly and portal hypertension (173).

The thymic cortex atrophied and the number of cortical thymocytes decreased in mice infected with *S. mansoni*, resulting in the loss of distinction in the corticomedullary region during the acute infection phase (98). *S. japonicum* can also induce thymic atrophy with the accumulation of both CD8+CD28- T cells and CD4+CD28- T cells (99). In addition, thymocytotoxic autoantibodies induced by *S. japonicum* were found in the thymus (100).

In summary, *T. Cruzi* is currently the most thoroughly studied parasite that causes thymic atrophy. The mechanisms of *T. Cruzi* -induced thymic atrophy mainly include apoptosis of thymocytes, premature exiting of thymocytes, alteration of the thymic microenvironment and regulation of hormones. So far, there are few studies on thymic atrophy caused by other parasites, and most of them focused on changes in thymus structure and changes in different thymocyte subpopulations, including cortical loss, CMJ loss and depletion of certain T cell subgroups (more details could be seen in **Table 3**). It needs further exploration in molecular mechanisms, changes of the thymic microenvironment and the interaction between the host and parasites. Besides, given that parasites are typical pathogens of chronic infection, more mechanisms of thymic atrophy induced by parasites need to be further studied, which is of great significance to study the damage of chronic infection to the body.

## Fungus

*Paracoccidioides brasiliensis* (*P. brasiliensis, Pb*) can invade the thymus causing severe thymic atrophy. Brito et al. (101) reported that after intraperitoneal injection of *P. brasiliensis*, the weight of the thymus decreased, the thymic cortex degenerated substantially and the CMJ disappeared. In the medullary region, *Pb* yeast cells were surrounded by histiocytes and there was predominant inflammatory infiltration of neutrophils and eosinophils in the medullary and subcapsular region. A subsequent study by Di Gangi et al. (102) showed that the spatial distribution of TECs was misplaced after *Pb* invasion of the thymus. The gene expression levels of IL-2, IL-7, IL-17, TNF-α and autoimmune regulator (AIRE) in the thymus increased significantly. *Pb*-induced thymic atrophy did not change the proportion of the various thymocyte subpopulations, that is, the cell number of all subpopulations decreased in the same proportion. This is different from the thymic atrophy characterized by significant reduction of DP cells caused by other pathogens. The increased expression of CCL19 and CCR7 in the thymus of infected mice indicated that the maturation of thymocytes was accelerated and the migration ability of thymocytes was stronger than that of the control group. In addition, autoreactive T cells that should have been eliminated during negative selection were found in peripheral lymphoid tissue, suggesting a potential risk of autoimmune disease after *Pb* infection. Costa et al. (103) established a model of chronic infection of *Pb* in mice. In the process of chronic infection, *Pb* severely infiltrated the thymus gland and granulomas were formed. In addition to the increased levels of IL-1β, IL-17, IL-18, IFN-γ and TNF-α in the thymus, high expression of caspase-1 and caspase-8 and the activation of inflammasome NLRP3 were also detected. Furthermore, an increase in the proportion of Th17 and CD8^+^ IFN-γ producing T cells in the thymus was detected, suggesting that mature T cells returned to the thymus to participate in the inflammatory response. Finally, apoptosis of thymocytes and thymic epithelial cells occurred, and the morphology and function of the thymus changed, forming the characteristics resembling secondary lymphoid tissues.

Some fungi release substances that mediate thymic atrophy. Gliotoxin produced by *Aspergillus fumigatus* has been proved to induce apoptosis of thymocytes *in vivo* (104). The T-2 toxin produced by the *Fusarium* species could also induce apoptosis of four thymocyte subsets (DN, DP, CD8^+^ CD4^-^, CD8^-^CD4^+^) *in vivo*, resulting in thymic atrophy, but this process did not involve regulation of glucocorticoid and TNF-α (105). In addition, there has been a report of thymic atrophy in fish caused by *Saprolegnia* (106). More information about fungi that induce thymic atrophy can be seen in **Table 4**.

## Discussion

Thymic atrophy induced by pathogens is a common phenomenon in infectious diseases. At the structural level, thymic atrophy caused by different pathogens presents certain similarities, but also has differences. In terms of structural alteration, the thymic cortex seems to be mainly affected by thymic atrophy. Depletion of thymocytes in the cortex can be observed in PRRSV, BVDV, SIV, MuLV, *Salmonella* Typhimurium, *Francisella tularensis* and many other pathogens. Accompanied by cortical involvement, CMJ also becomes obscure. Additionally, there are other histological changes that can be observed, including collagen deposition in BVDV (26) and congestion in (AIV)-H9N2 (33).

**Figure 1:**
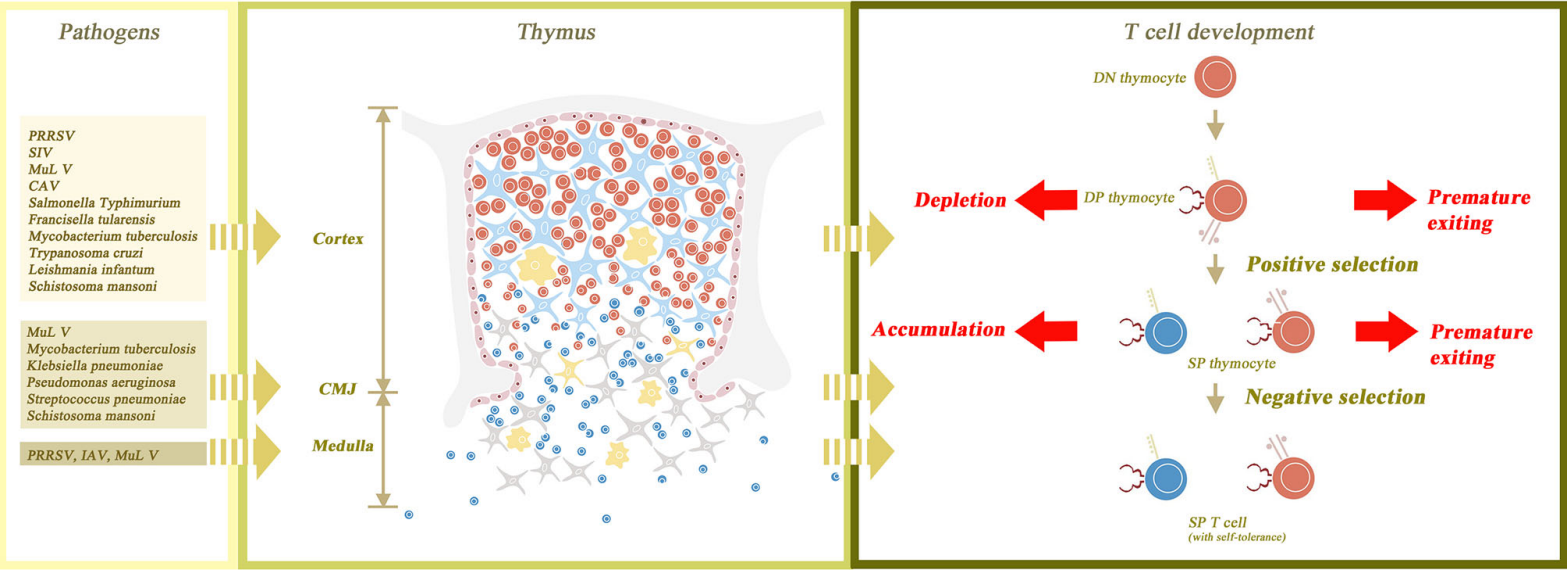
The normal structure of the thymus gland and the changes that occur with atrophy. The thymus parenchyma is composed of cortex and medulla, which are separated by cortico-medullary junction (CMJ). The cortex is constructed from thymic epithelial cells (TEC) as its framework and thymocytes filling in the interspace. The medulla is composed of TECs, naïve T cells and macrophages. The process of T cell maturation begins with the transformation of double negative (DN) thymocytes to double positive (DP) thymocytes in the outer cortex of the thymus gland. DP thymocytes then undergo positive and negative selection in the inner cortex and medulla, respectively, to become self-tolerant single positive (SP) thymocytes. Finally, SP thymocytes mature and are transported to peripheral lymphoid tissues. Thymic atrophy leads to the destruction of thymus structure and abnormal maturation of thymocytes. In thymic atrophy, the medullary structure is destroyed and the CMJ is disappeared, so both positive and negative selection are interrupted. Without positive selection, premature exiting of DP thymocytes may occur. Without negative selection, the autoreactive T cells may have the opportunity to migrate out of the thymus. In addition, cortical structural disorders not only cause the depletion of a large number of DP thymocytes, but also delay the maturation of SP thymocytes, leading to the accumulation of SP thymocytes. Besides, the figure also shows the main affected sites of thymus corresponding to different pathogens.

The thymus is the specific site for T cell maturation, and therefore, thymic atrophy will lead to a series of dysfunctions in the thymocyte maturation process. Positive selection occurs in the inner cortex whereas negative selection occurs in the medulla. The cortex and (occasionally) the medulla are structurally damaged in infection-induced thymic atrophy, thus interrupting both positive and negative selection. Failing to undergo positive selection, a premature exit of DP thymocytes is observed in MDV (56), *Trypanosoma cruzi* (85, 86) and *Plasmodium berghei* (91, 92). Disordered cortical structure leads to delayed maturation of SP thymocytes, which results in the accumulation of CD24^high^CD3^high^ SP cells. This phenomenon is observed in *Salmonella Typhimurium* infection (62). Skipping the process of negative selection, T cells become auto-reactive. *T. cruzi* infection-induced myocarditis may be associated with it (148). Such auto-immune diseases have also been reported in *P. brasiliensis* infection (102). As for the functional alteration of infection-induced thymic atrophy, double positive (DP) thymocytes are the general cluster of depletion. A recent study shows that auto-immunity facilitates the death of thymic epithelial cells through returning Th1 cells (174), which reveals that auto-immune disease and thymic atrophy may be linked in a vicious cycle, leading to the disorder of immune tolerance. Double negative (DN) thymocytes are the main apoptotic cluster although other clusters are involved as well. CD4+ SP thymocytes are depleted in *Plasmodium yoelii* infected models (95). Both CD4+ and CD8+ SP thymocytes, as well as δγ+ T cells, are depleted in *Plasmodium chabaudi* infected models (94). The structure of the thymus and the changes in its composition due to thymic atrophy are shown in **Figure 1**. Recently, novel research sought to determine subpopulations of thymic atrophy under different insults, including injection with *Salmonella Typhimurium*, lipopolysaccharide (LPS) and two other non-infectious insults (etoposide and dexamethasone). In this research, all these insults led to depletion of DP cells. However, DN cells and CD8^+^CD3^lo^ immature SP cells were only vulnerable to infection, LPS and etoposide, but not to dexamethasone. The mature SP thymocytes were reduced by the insults of etoposide and dexamethasone, but infection did not affect this process (175). Although the link between the mechanism and target subpopulation of thymic atrophy is not yet clear, researchers are certain that different pathogens induce thymic atrophy in different ways.

Atrophy is defined as shrinkage of the volume of a parenchyma together with decrease of the number of parenchyma cells. Cell death occurs through different mechanisms, including necrosis (passive and uncontrolled cell death) and apoptosis (active and programmed cell death) (176). Apoptosis is the main mechanism of infection-induced thymic atrophy. For example, apoptosis of CD3+ thymocyte is observed in PRRSV (21). Necrosis also plays a small role in infection-induced thymic atrophy, which is reported in NDV, *F. tularensis* and administration of cholera toxin (CT) or heat-labile enterotoxin (LT) (78, 121, 122). It should be noted that other types of cellular death are involved in thymic atrophy. Inflammasome activity and gene expression of caspase-1 in *Paracoccidioides brasiliensis* indicates that pyroptosis participates in the induction of thymic atrophy (103). Autophagy, a kind of multistep lysosomal degradation pathway, is observed in the HP-PRRSV (HuN4) infection model (23).

**Figure 2:**
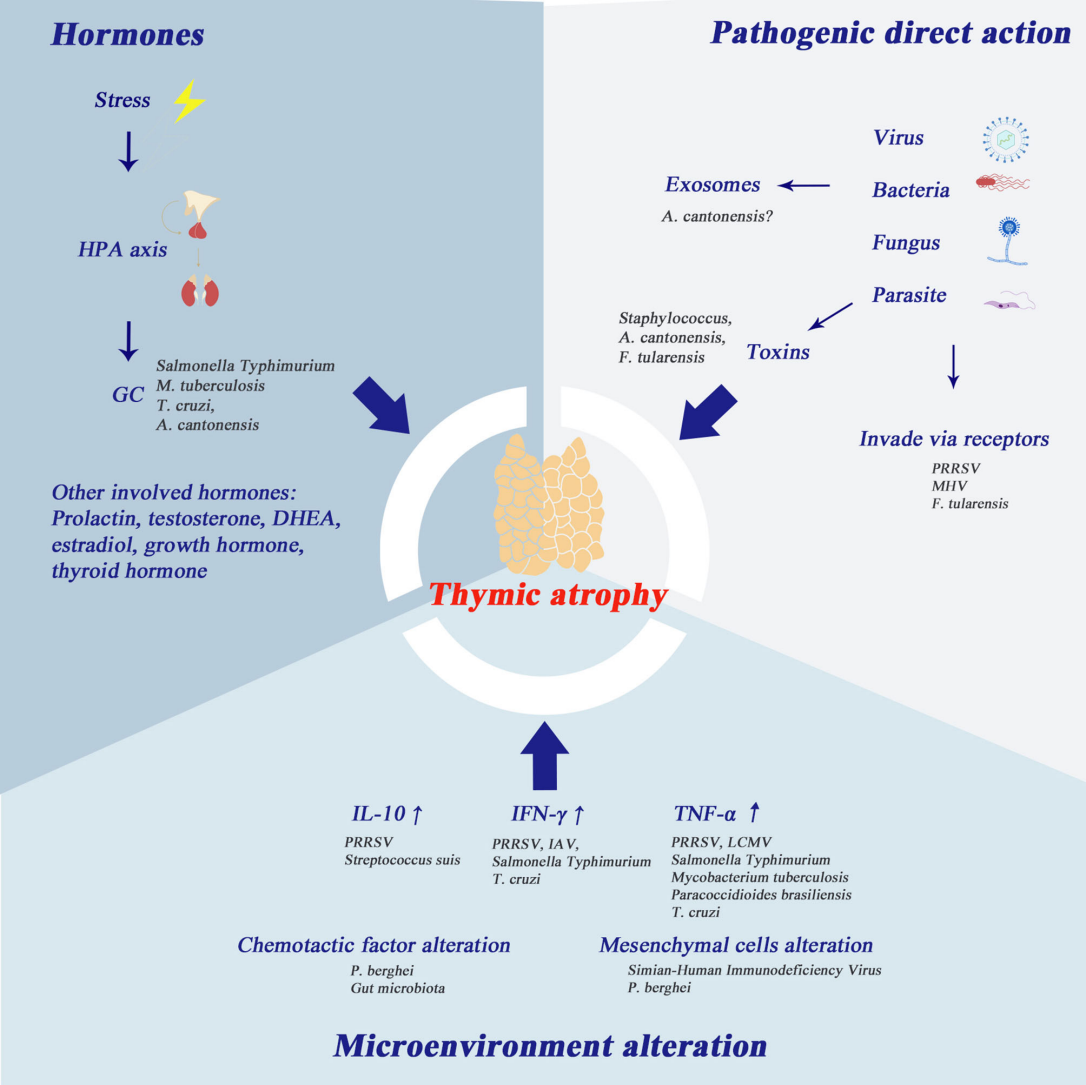
Mechanisms of infection-induced thymic atrophy. This figure summarizes the three main mechanisms of infection-related thymic atrophy and lists the characteristic pathogens corresponding to each mechanism. 1) The hypothalamic-pituitary-adrenal (HPA) axis plays an important role in infection-induced thymic atrophy. Glucocorticoids can induce apoptosis of thymocytes, especially DP thymocytes. In addition, other hormones mentioned in the picture are also thought to be involved in thymic atrophy. 2) At the molecular level, the change of thymus microenvironment is one of the major changes associated with thymic atrophy, and the main cytokines involved are IL-10, IFN-γ and TNF-α. 3) Some pathogens take the thymus as the target organ and directly invade the thymus. In addition, the products of pathogens, such as toxins, soluble antigens, and exosomes, can also play an important role in thymic atrophy.

Although many pathogens can cause thymic atrophy, the mechanisms through which they fulfill this process are quite different. To date, many mechanisms have been demonstrated to be involved in this pathogenic process and conclusions in this regard are shared in **Figure 2**.

Glucocorticoid secretion is a responsive effect initiated by stress. It has been reported in other models that corticoids can cause lymphocyte depletion. Compared with osteopontin (OPN) (-/-) mice, OPN (+/+) mice showed thymic and splenic atrophy by modulation of corticosterone *via* the OPN signaling pathway (177). Glucocorticoid hormone level is also thought to be related to infection-induced thymic atrophy. Steroids can trigger apoptosis in thymocytes, in which DP thymocytes are the most sensitive to glucocorticoids (18). This could easily explain why the phenotype of the major apoptotic cluster is CD4+CD8+ thymocytes. SP thymocytes rely on CD28 signaling to be more resistant to glucocorticoid-induced apoptosis (18). In several bacteria-associated infections, such as sepsis, *Salmonella Typhimurium* and tuberculosis, elevation in glucocorticoids is observed. In Chagas disease, glucocorticoids induce thymic atrophy, which can be countered by dehydroepiandrosterone (155, 156) and prolactin (154). The HPA axis has been reported to participate in *Angiostrongylus cantonensis* induced thymic atrophy (96).

The thymic micro-environment alteration is one of the main alterations at molecular level that accompanies thymic atrophy. Elevation of IL-10 was observed in PRRSV and *Streptococcus suis*, while elevation of IFN-γ was observed in PRRSV, IAV, *Salmonella* Typhimurium and *Trypanosoma cruzi.* IL-10 elevation is associated with thymic atrophy, while neem leaf glycoprotein (NLGP) can reverse tumor- and age-associated thymic atrophy through downregulating IL-10 (178). Type I IFN in chronic LCMV infection has been shown to trigger severe thymic depletion through signal transducers and activators of transcription 2 (Stat2) signaling (34). TNF-α is elevated in infections of PRRSV, LCMV, *Salmonella* Typhimurium and bacteria targeting the respiratory system, e.g., *Mycobacterium tuberculosis. Trypanosoma cruzi* and *Paracoccidioides brasiliensis* also lead to elevation of TNF-α. In the *Plasmodium berghei* infection model and the CLP model, the alterations of chemotactic factors and chemotactic factor receptors have been reported to be the main changes in the thymic microenvironment. Because the interactions between chemotactic factor ligands and their receptors play an important role in T-cell maturation, location and homing, the alterations of these interactions lead to T cell dysfunction. Some pathogen-induced thymic atrophy causes alteration of mesenchymal and parenchymal cells. Increased expression of ECM component is detected in *Plasmodium berghei* infection. Destruction of thymic lobar tissue (and replacement of connective tissue) is observed in the infection of Simian-Human Immunodeficiency Virus. Mesenchymal cells function as the regulator of thymocyte location, adhesion and migration. The dysfunction of mesenchymal cells results in a disordered pattern of thymocyte arrangement.

In addition to changes in the thymus microenvironment (i.e. pathogens do not directly attack the thymus), many other pathogens directly target the thymus. PRRSV invades the thymus directly. However, infected cells are depleted *via* autophagy not apoptosis (23). In MHV infection models, thymocytes replicating virus nucleotides are depleted in a manner of apoptosis (51). Methods to verify that the thymus is the target organ of certain viruses are either through demonstrating the replicating phenomena in the thymus or through purifying the receptors for viral invasion from the thymus. Some scientists use toxins or substances produced from pathogens instead of pathogens themselves for experiments. Interestingly, many components of a pathogen can induce thymic atrophy alone. Lin et al. (77) noted that staphylococcal enterotoxin B (SEB) can lead to thymocyte apoptosis. Similarly, Liu et al. (97) observed that soluble antigen from *A. cantonensis* can lead to apoptosis of thymic stromal cells and thymocytes. In addition, *F. tularensis* is also found in thymus tissue during infection (78).

Current studies of exosomes have shown that these small vesicles secreted by living cells, have the function of targeting the transport of substances in the vesicles, such as protein, DNA and lncRNA, to specific tissue cells. Exosomes thus play an important role in the information exchange between cells (179). They may be a more effective mediator in the interaction between pathogens and hosts, rather than soluble antigens produced by pathogens. Recent studies show that exosomes can carry bioactive substances and fuse with the host target cells, thus regulating the immune response of the host (180, 181) and participating in the pathogenic pathological process (182). Twu et al. found that exosomes originating from *Trichomonas vaginalis* contained surface proteins and proteases, potentially involved in pathogenesis. This exosome fused with and delivered its content to ectocervical cells, contributing to the adhesion and pathogenesis of *Trichomonas vaginalis* (183). In a similar fashion, exosomes from *Trypanosoma* were also found to mediate release of various proteins including some proteases, whose functions include promoting invasion, protecting *Trypanosoma* from host immunity and affecting physiology of host cells (184, 185). Additionally, RNA in exosomes may also play a non-negligible role here. Zhu et al. found that exosomes derived from *Schistosoma japonicum* and their cargo mi-RNA could be internalized by mammalian cells (186). Exosomes derived from *Trypanosoma cruzi* can modify some genes of host cells *via* abundant tRNA-derived small RNAs (187). Exosomes originating from pathogens possess immune-stimulatory or inhibitory effects. Wang et al. demonstrated that exosomes originating from *S. japonicum* promoted M1 macrophage polarization and release of pro-inflammatory factors (188). Oliveira et al. found that incubation of *Cryptococcus neoformans* vesicles with murine macrophages resulted in increased levels of TNF-alpha, IL-1, and TGF-beta, and stimulated nitric oxide production by phagocytes thus enhancing antimicrobial function (189). Buck et al. discovered that exosomes from *Heligmosomoides polygyrus* suppressed type 2 innate responses. In mouse cells that are incubated with *H. polygyrus* exosomes, Dusp1, an attenuator of immune activation, is suppressed by miRNA (190). Increasing research evidence indicates the close and complex relationship between host and exosomes originating from pathogens. Interestingly, it is mentioned above that the administration of the soluble antigen of *A. cantonensis* into the thymus of mice can cause obvious thymic atrophy, as for mice directly infected with *A. cantonensis* (97). Given that soluble antigens of *A. cantonensis* may break down during a long journey from the brain to the thymus, we reasonably speculate that the transport of soluble antigens should rely on a more effective vector, with exosomes the suitable choice. Therefore, it is reasonable to infer that some pathogenic products released by pathogens can be precisely transported to the thymus *in vivo* by means of exosomes, thus leading to apoptosis of thymic cells and TECs. However, to date this is mere speculation and requires further verification by experiments.

Advances in studies of the mechanisms underlying thymic atrophy have led to subsequent proposals about its reversal. Different therapies such as nutritional supplementation (i.e. Zinc, antioxidants), cytokines, chemokines, hormones, and growth factors have been reported to be effective in reversing thymic atrophy (191). However, for infection-induced thymic atrophy, there is less related research at present. Therapeutic methods for infection-induced thymic atrophy are not fully established. In the studies that have been done so far, it is reported that in HIV patients, high dose treatment of N-acetyl cysteine (a prodrug of glutathione) and vitamin C could induce higher numbers of CD4+ T cells (192). Leptin attenuates LPS-induced thymocyte apoptosis by down regulating cPLA2 and p38 MAPK activation (193). In *T. cruzi*-infected mice, melatonin reverts thymic atrophy by increasing thymic weight, thymocyte total number and the ratio of DP thymocytes (194). During infections of pathogens, like *T. cruzi*, glucocorticoids play a vital role in thymic atrophy *via* activating caspase 8 and caspase 9. The administration of steroid receptor RU486 can reverse this effect of GC in *T. cruzi-*infected mice (149). However, it cannot be ignored that GC has a protective effect on the host during infection, as the blocking of GC receptor RU486 accelerates host death, which has been demonstrated experimentally (150). Another stress-induced hormone, prolactin, is also known to restrict GC-induced thymic atrophy and can export immature DP thymocytes to the periphery (154). The question of what hormone we can use to manually intervene, and how to regulate them to produce maximum therapeutic results, safely for patients, is worthy of more research. Exosomes may offer distinct potential to reverse thymic atrophy. Recent research has demonstrated that extracellular vesicles isolated from plasma of young mice could increase the longevity and delay inflammaging of old mice (195, 196). Wnt4-transgenic TEC-derived exosomes demonstrated the ability to delay age-related thymic atrophy (197). Moreover, exosomes or extracellular vesicles extracted from juvenile mice could reverse thymic atrophy and decrease the autoimmune reactions (196), indicating that exosome injection as a therapy could be an option. Exosomes produced by pathogens may be used as carriers of pathogenic substances. Thus, it is reasonable to speculate that exosomes in normal human or mouse serum may contain substances that can ameliorate thymic atrophy. Purified normal serum exosomes may provide a new solution for the treatment of thymic atrophy in the future.

To date, limited studies have been conducted on infection-induced thymic atrophy in humans, and more mechanisms need to be discovered to update existing theories. At present, all studies on the mechanism of infection-induced thymic atrophy focuses on the changes of the thymic microenvironment. And these studies attempted to explain the possible causes of thymic atrophy through the changes in cytokines and hormones in body fluid, while ignoring the possible role of neuromodulation. The regulation of homeostasis in the human body is achieved by the neuro-humoral-immune regulation network. Pathogen infection, as a stressor, will cause a strong stress response, which inevitably involves neuromodulation. The thymus is innervated by both sympathetic and parasympathetic nerves (198). Under long-term stress, sympathetic nerve excitation will make peripheral blood vessels contract violently for a long time to distribute more blood to important organs such as the liver and brain. This may lead to insufficient blood supply to the thymus and induce apoptosis or necrosis. Furthermore, studies have confirmed that the proliferation and function of T cells will be inhibited under the condition of high concentration of catecholamines (199). The sympathetic nervous system is also closely related to the function of the HPA axis under stress (200), suggesting that it may be involved in the HPA-related mechanism of thymic atrophy. In addition, the thymus is innervated by the non-adrenergic and non-cholinergic (NANC) nervous system. In the case of severe infection such as sepsis, NANC releases substance P and neurokinin A to increase vascular permeability and inflammatory exudation (201), which may aggravate infection-induced thymic atrophy when these occur in the thymus. It is possible that there are potentially more neuro-related mechanisms to be discovered. Therefore, we suggest that researchers devote more attention to exploring the effects of neuroregulatory mechanisms on infection-induced thymic atrophy in future.

With medical advances, most infections can be controlled in the acute phase. However, chronic infections, often ignored by both doctors and patients, can also have a serious impact on the body, especially in children, whose thymic atrophy may occur ahead of time in the process of chronic infection. For example, chronic oral infections, such as dental caries, gingivitis and periodontitis, are demonstrated to be related to diabetes and preterm birth (202), and immune disorders are involved in their pathogenesis. Periodontitis can lead to type 1 diabetes by mediating decrease in insulin release and insulin resistance through cytokines alteration (203). Moreover, in children, chronic infections may affect the developing repertoire of T cells (6). Thus, it’s necessary to pay more attention to the residual chronic infection while controlling the acute infection. And it is of great significance to enhance the ability to detect the micro-infection in the field of clinical diagnosis and testing. Under chronic infection, the pathogen interacts with the thymus through the mechanisms mentioned above to induce thymic atrophy, and then leads to a series of immune disorders. By summarizing these mechanisms, we hope to provide inspiration for the selection of predictors for early diagnostic models of chronic infection in the future.

As noted above, there are both similarities and differences in the pathologic manifestations of infection-related and age-related thymic atrophy. Although the mechanism of their occurrence is slightly different at present, whether there is a common downstream pathway is worth further investigation. Therefore, our summary of the mechanisms of infection-induced thymic atrophy may also suggest a potential mechanism for age-related thymic atrophy. We also summarize possible measures to reverse infection-related thymic atrophy, we hope that researchers will take inspiration from these treatments that target different mechanisms and one day find a way to reverse age-related thymic atrophy and boost the body’s immune system.

## Author Contributions

ML, LX, and ZQ have contributed equally to this work and share first authorship. XS is the corresponding author. All authors contributed to the article and approved the submitted version.

## Conflict of Interest

The authors declare that the research was conducted in the absence of any commercial or financial relationships that could be construed as a potential conflict of interest.

## References

[1] Kindt TJ Goldsby RA Osborne BA Kuby J. Kuby Immunology (2007). Available at: https://books.google.com.hk/books?id=oOsFf2WfE5wC.

[2] Gascoigne NR Rybakin V Acuto O Brzostek J. Tcr Signal Strength and T Cell Development. Annu Rev Cell Dev Biol (2016) 32:327–48. 10.1146/annurev-cellbio-111315-125324 27712102

[3] Cepeda S Griffith A. Thymic Stromal Cells: Roles in Atrophy and Age-Associated Dysfunction of the Thymus. Exp Gerontol (2018) 105:113–7. 10.1016/j.exger.2017.12.022 PMC5869099 29278750

[4] Fraker P King L. Reprogramming of the Immune System During Zinc Deficiency. Annu Rev Nutr (2004) 24:277–98. 10.1146/annurev.nutr.24.012003.132454 15189122

[5] Savino W Dardenne M. Nutritional Imbalances and Infections Affect the Thymus: Consequences on T-cell-mediated Immune Responses. Proc Nutr Soc (2010) 69(4):636–43. 10.1017/s0029665110002545 20860857

[6] Nunes-Alves C Nobrega C Behar SM Correia-Neves M. Tolerance has its Limits: How the Thymus Copes With Infection. Trends Immunol (2013) Oct 34(10):502–10. 10.1016/j.it.2013.06.004 PMC3879077 23871487

[7] Aspinall R Andrew D. Thymic Involution in Aging. J Clin Immunol (2000) 20(4):250–6. 10.1023/a:1006611518223 10939712

[8] Carrio R Torroella-Kouri M Iragavarapu-Charyulu V Lopez DM. Tumor-Induced Thymic Atrophy: Alteration in Interferons and Jak/Stats Signaling Pathways. Int J Oncol (2011) 38(2):547–53. 10.3892/ijo.2010.870 21165556

[9] Thomas R Wang W Su DM. Contributions of Age-Related Thymic Involution to Immunosenescence and Inflammaging. Immun Ageing (2020) 17:2. 10.1186/s12979-020-0173-8 31988649 PMC6971920

[10] Gruver A Hudson L Sempowski G. Immunosenescence of Ageing. J Pathol (2007) 211(2):144–56. 10.1002/path.2104 PMC1931833 17200946

[11] O’Sullivan BJ Yekollu S Ruscher R Mehdi AM Maradana MR Chidgey AP. Autoimmune-Mediated Thymic Atrophy Is Accelerated But Reversible in RelB-Deficient Mice. Front Immunol (2018) 9:1092. 10.3389/fimmu.2018.01092 29872433 PMC5972300

[12] Aspinall R Andrew D. Immunosenescence: Potential Causes and Strategies for Reversal. Biochem Soc Trans (2000) 28(2):250–4. 10.1042/bst0280250 10816137

[13] Rehman S Majeed T Azam Ansari M Ali U Sabit H Al-Suhaimi E. Current Scenario of COVID-19 in Pediatric Age Group and Physiology of Immune and Thymus Response. Saudi J Biol Sci (2020) 27(10):2567–73. 10.1016/j.sjbs.2020.05.024 PMC7227606 32425651

[14] Lins MP Smaniotto S. Potential Impact of SARS-CoV-2 Infection on the Thymus. Can J Microbiol (2021) 67(1):23–8. 10.1139/cjm-2020-0170%M32640169 32640169

[15] Sato Y Yanagita M. Immunology of the Ageing Kidney. Nat Rev Nephrol (2019) 15(10):625–40. 10.1038/s41581-019-0185-9 31477915

[16] Nikolich-Žugich J. The Twilight of Immunity: Emerging Concepts in Aging of the Immune System. Nat Immunol (2018) 19(1):10–9. 10.1038/s41590-017-0006-x 29242543

[17] Palmer S Albergante L Blackburn CC Newman TJ. Thymic Involution and Rising Disease Incidence With Age. Proc Natl Acad Sci U S A (2018) 115(8):1883–8. 10.1073/pnas.1714478115 PMC5828591 29432166

[18] Savino W. The Thymus is a Common Target Organ in Infectious Diseases. PloS Pathog (2006) 2(6):e62. 10.1371/journal.ppat.0020062 16846255 PMC1483230

[19] Aspinall R. Age-Associated Thymic Atrophy in the Mouse is Due to a Deficiency Affecting Rearrangement of the TCR During Intrathymic T Cell Development. J Immunol (1997) Apr 1 158(7):3037–45. 9120255

[20] Tyan ML. Age-Related Decrease in Mouse T Cell Progenitors. J Immunol (1977) 118(3):846–51. 300390

[21] Amarilla SP Gomez-Laguna J Carrasco L Rodriguez-Gomez IM Caridad YOJ Graham SP. Thymic Depletion of Lymphocytes is Associated With the Virulence of PRRSV-1 Strains [Journal Article]. Vet Microbiol (2016) 188:47–58. 10.1016/j.vetmic.2016.04.005%/ Copyright (c) 2016 Elsevier B.V 27139029

[22] He Y Wang G Liu Y Shi W Han Z Wu J. Characterization of Thymus Atrophy in Piglets Infected With Highly Pathogenic Porcine Reproductive and Respiratory Syndrome Virus. Vet Microbiol (2012) 160(3-4):455–62. 10.1016/j.vetmic.2012.05.040 22763175

[23] Wang G Yu Y Tu Y Tong J Liu Y Zhang C. Highly Pathogenic Porcine Reproductive and Respiratory Syndrome Virus Infection Induced Apoptosis and Autophagy in Thymi of Infected Piglets. PloS One (2015) 10(6):e0128292. 10.1371/journal.pone.0128292 26046751 PMC4457848

[24] Wang G Song T Yu Y Liu Y Shi W Wang S. Immune Responses in Piglets Infected With Highly Pathogenic Porcine Reproductive and Respiratory Syndrome Virus. Vet Immunol Immunopathol (2011) 142(3-4):170–8. 10.1016/j.vetimm.2011.05.004 21612828

[25] Ruedas-Torres I Rodríguez-Gómez IM Sánchez-Carvajal JM Pallares FJ Barranco I Carrasco L. Activation of the Extrinsic Apoptotic Pathway in the Thymus of Piglets Infected With PRRSV-1 Strains of Different Virulence. Vet Microbiol (2020) 243:108639. 10.1016/j.vetmic.2020.108639 32273018

[26] Romero-Palomo F Risalde MA Molina V Lauzi S Bautista MJ Gomez-Villamandos JC. Characterization of Thymus Atrophy in Calves With Subclinical BVD Challenged With BHV-1. Vet Microbiol (2015) 177(1-2):32–42. 10.1016/j.vetmic.2015.02.018. Cited in: Pubmed 25759294

[27] M K K T M V T HJ R T. CD46 is a Cellular Receptor for Bovine Viral Diarrhea Virus. J Virol (2004) 78(4):1792–9. 10.1128/jvi.78.4.1792-1799.2004 PMC369467 14747544

[28] Duan X Lu J Zhou K Wang J Wu J Gao GF. NK-Cells are Involved in Thymic Atrophy Induced by Influenza A Virus Infection. J Gen Virol (2015) 96(11):3223–35. 10.1099/jgv.0.000276 26346306

[29] Silvano FD Kanata Y Takeuchi M Shimada A Otsuki K Umemura T. Avian Influenza A Virus Induced Stunting Syndrome-Like Disease in Chicks. J Vet Med Sci (1997) 59(3):205–7. 10.1292/jvms.59.205 9101480

[30] Liu B Zhang X Deng W Liu J Li H Wen M. Severe Influenza A(H1N1)pdm09 Infection Induces Thymic Atrophy Through Activating Innate CD8(+)CD44(Hi) T Cells by Upregulating IFN-Gamma. Cell Death Dis (2014) 5:e1440. 10.1038/cddis.2014.323 25275588 PMC4649502

[31] Berg LJ. Signalling Through TEC Kinases Regulates Conventional Versus Innate CD8(+) T-Cell Development. Nat Rev Immunol (2007) 7(6):479–85. 10.1038/nri2091 17479128

[32] Liu B Bao L Wang L Li F Wen M Li H. Anti-IFN-γ Therapy Alleviates Acute Lung Injury Induced by Severe Influenza A (H1n1) pdm09 Infection in Mice. J Microbiol Immunol Infect (2019) S1684-1182(18):30438–9. 10.1016/j.jmii.2019.07.009. Cited in: Pubmed 31780358

[33] Hassan KE Ali A Shany SAS El-Kady MF. Experimental Co-Infection of Infectious Bronchitis and Low Pathogenic Avian Influenza H9N2 Viruses in Commercial Broiler Chickens. Res Vet Sci (2017) 115:356–62. 10.1016/j.rvsc.2017.06.024 PMC7172277 28692924

[34] Elsaesser HJ Mohtashami M Osokine I Snell LM Cunningham CR Boukhaled GM. Chronic Virus Infection Drives CD8 T Cell-Mediated Thymic Destruction and Impaired Negative Selection. Proc Natl Acad Sci U S A (2020) 117(10):5420–9. 10.1073/pnas.1913776117 PMC7071912 32094187

[35] Orange JS Salazar-Mather TP Opal SM Spencer RL Miller AH McEwen BS. Mechanism of Interleukin 12-Mediated Toxicities During Experimental Viral Infections: Role of Tumor Necrosis Factor and Glucocorticoids. J Exp Med (1995) 181(3):901–14. 10.1084/jem.181.3.901 PMC2191920 7869050

[36] Muller JG Krenn V Czub S Stahl-Hennig C Coulibaly C Hunsmann G. The Thymic Epithelial Reticulum and Interdigitating Cells in SIV-induced Thymus Atrophy and its Comparison With Other Forms of Thymus Involution. Res Virol (1993) 144(1):93–8. 10.1016/s0923-2516(06)80017-8 8446784

[37] Sodora D Milush J Ware F Wozniakowski A Montgomery L McClure H. Decreased Levels of Recent Thymic Emigrants in Peripheral Blood of Simian Immunodeficiency Virus-Infected Macaques Correlate With Alterations Within the Thymus. J Virol (2002) 76(19):9981–90. 10.1128/jvi.76.19.9981-9990.2002 PMC136511 12208974

[38] Stephens EB McCormick C Pacyniak E Griffin D Pinson DM Sun F. Deletion of the Vpu Sequences Prior to the Env in a Simian-Human Immunodeficiency Virus Results in Enhanced Env Precursor Synthesis But is Less Pathogenic for Pig-Tailed Macaques [Comparative Study; Journal Article; Research Support, U.S. Gov’t, P.H.s.]. Virology (2002) 293(2):252–61. 10.1006/viro.2001.1244 11886245

[39] Fiume G Scialdone A Albano F Rossi A Tuccillo FM Rea D. Impairment of T Cell Development and Acute Inflammatory Response in HIV-1 Tat Transgenic Mice. Sci Rep (2015) 5:13864. 10.1038/srep13864 26343909 PMC4561375

[40] Tesselaar K Miedema F. Growth Hormone Resurrects Adult Human Thymus During HIV-1 Infection. J Clin Invest (2008) 118(3):844–7. 10.1172/jci35112 PMC2248805 18292816

[41] Diehl LJ Mathiason-Dubard CK O’Neil LL Obert LA Hoover EA. Induction of Accelerated Feline Immunodeficiency Virus Disease by Acute-Phase Virus Passage. J Virol (1995) 69(10):6149–57. 10.1128/JVI.69.10.6149-6157.1995 PMC189512 7666517

[42] Johnson CM Bortnick SJ Crawford PC Papadi GP. Unique Susceptibility of the Fetal Thymus to Feline Immunodeficiency Virus Infection: An Animal Model for HIV Infection In Utero. Am J Reprod Immunol (2001) 45(5):273–88. 10.1111/j.8755-8920.2001.450503.x 11432402

[43] Hayasaka D Ennis FA Terajima M. Pathogeneses of Respiratory Infections With Virulent and Attenuated Vaccinia Viruses. Virol J (2007) 4:22. 10.1186/1743-422x-4-22 17326843 PMC1810241

[44] R PC M JB S GL. Steroid Hormone Synthesis by Vaccinia Virus Suppresses the Inflammatory Response to Infection. J Exp Med (2003) 197(10):1269–78. 10.1084/jem.20022201 PMC2193778 12756265

[45] Chen X Da R Jin X Song W Li X Fu Y. Cross-Species Infectivity and Pathogenesis of the Friend Murine Leukemia Virus Complex in Syrian Hamsters. Virus Res (2007) 130(1-2):281–4. 10.1016/j.virusres.2007.05.015 17602778

[46] Bonzon C Fan H. Moloney Murine Leukemia Virus-Induced Preleukemic Thymic Atrophy and Enhanced Thymocyte Apoptosis Correlate With Disease Pathogenicity. J Virol (1999) 73(3):2434–41. 10.1128/JVI.73.3.2434-2441.1999 PMC104490 9971828

[47] de Leval L Deprez M Colombi S Humblet C Defresne MP Boniver J. Morphological Changes of Thymus in Retrovirus-Induced Murine Acquired Immunodeficiency Syndrome (MAIDS). Pathol Res Pract (1995) 191(6):506–12. 10.1016/s0344-0338(11)80869-1 7479371

[48] Haridy M Sasaki J Ikezawa M Okada K Goryo M. Pathological and Immunohistochemical Studies of Subclinical Infection of Chicken Anemia Virus in 4-Week-Old Chickens. J Vet Med Sci (2012) 74(6):757–64. 10.1292/jvms.11-0374 22293470

[49] Castano P Benavides J Lee MS Fernandez M Fuertes M Royo M. Tissue Tropism of Chicken Anaemia Virus in Naturally Infected Broiler Chickens. J Comp Pathol (2019) 167:32–40. 10.1016/j.jcpa.2018.11.008 30898295

[50] Shawky S Sandhu T Shivaprasad HL. Pathogenicity of a Low-Virulence Duck Virus Enteritis Isolate With Apparent Immunosuppressive Ability. Avian Dis (2000) 44(3):590–9. 10.2307/1593098 11007006

[51] Godfraind C Holmes KV Coutelier JP. Thymus Involution Induced by Mouse Hepatitis Virus A59 in BALB/c Mice. J Virol (1995) 69(10):6541–7. 10.1128/JVI.69.10.6541-6547.1995 PMC189556 7666556

[52] Munoz-Gonzalez S Ruggli N Rosell R Perez LJ Frias-Leuporeau MT Fraile L. Postnatal Persistent Infection With Classical Swine Fever Virus and its Immunological Implications. PloS One (2015) 10(5):e0125692. 10.1371/journal.pone.0125692 25938664 PMC4418595

[53] Fu Q Huang Y Wan C Fu G Qi B Cheng L. Genomic and Pathogenic Analysis of a Muscovy Duck Parvovirus Strain Causing Short Beak and Dwarfism Syndrome Without Tongue Protrusion. Res Vet Sci (2017) 115:393–400. 10.1016/j.rvsc.2017.07.006 28715672

[54] Ezema WS Eze DC Shoyinka SV Okoye JO. Atrophy of the Lymphoid Organs and Suppression of Antibody Response Caused by Velogenic Newcastle Disease Virus Infection in Chickens. Trop Anim Health Prod (2016) 48(8):1703–9. 10.1007/s11250-016-1147-x 27645826

[55] Okpe GC Ezema WS Shoyinka SV Okoye JO. Vitamin A Dietary Supplementation Reduces the Mortality of Velogenic Newcastle Disease Significantly in Cockerels. Int J Exp Pathol (2015) 96(5):326–31. 10.1111/iep.12138 PMC4693551 26511428

[56] Berthault C Larcher T Hartle S Vautherot JF Trapp-Fragnet L Denesvre C. Atrophy of Primary Lymphoid Organs Induced by Marek’s Disease Virus During Early Infection is Associated With Increased Apoptosis, Inhibition of Cell Proliferation and a Severe B-Lymphopenia. Vet Res (2018) 49(1):31. 10.1186/s13567-018-0526-x 29587836 PMC5870490

[57] Perry L Hotchkiss J Lodmell D. Murine Susceptibility to Street Rabies Virus is Unrelated to Induction of Host Lymphoid Depletion. J Immunol (Baltimore Md 1950) (1990) 144(9):3552–7. 2329281

[58] Cardenas Palomo L de Souza Matos D Chaves Leal E Bertho A Marcovistz R. Lymphocyte Subsets and Cell Proliferation Analysis in Rabies-Infected Mice. J Clin Lab Immunol (1995) 46(2):49–61. 8789128

[59] Yang D Zhao C Zhang M Zhang S Zhai J Gao X. Changes in Oxidation-Antioxidation Function on the Thymus of Chickens Infected With Reticuloendotheliosis Virus. BMC Vet Res (2020) 16(1):483. 10.1186/s12917-020-02708-6 33308224 PMC7731740

[60] Baily JL Willoughby K Maley M Chapman J Pizzi R Hall AJ. Widespread Neonatal Infection With Phocid Herpesvirus 1 in Free-Ranging and Stranded Grey Seals Halichoerus Grypus. Dis Aquat Organ (2019) 133(3):181–7. 10.3354/dao03345 31019131

[61] Deobagkar-Lele M Chacko SK Victor ES Kadthur JC Nandi D. Interferon-Gamma- and Glucocorticoid-Mediated Pathways Synergize to Enhance Death of CD4(+) CD8(+) Thymocytes During Salmonella Enterica Serovar Typhimurium Infection. Immunology (2013) 138(4):307–21. 10.1111/imm.12047 PMC3719942 23186527

[62] Majumdar S Deobagkar-Lele M Adiga V Raghavan A Wadhwa N Ahmed SM. Differential Susceptibility and Maturation of Thymocyte Subsets During Salmonella Typhimurium Infection: Insights on the Roles of Glucocorticoids and Interferon-Gamma. Sci Rep (2017) 7:40793. 10.1038/srep40793. Cited in: Pubmed 28091621 PMC5238503

[63] Deobagkar-Lele M Victor ES Nandi D. C-Jun NH2 -Terminal Kinase is a Critical Node in the Death of CD4+ CD8+ Thymocytes During Salmonella Enterica Serovar Typhimurium Infection. Eur J Immunol (2014) 44(1):137–49. 10.1002/eji.201343506 24105651

[64] Huang H Liu A Wu H Ansari AR Wang J Huang X. Transcriptome Analysis Indicated That Salmonella Lipopolysaccharide-Induced Thymocyte Death and Thymic Atrophy Were Related to TLR4-FOS/JUN Pathway in Chicks. BMC Genomics (2016) 17:322. 10.1186/s12864-016-2674-6 27142675 PMC4855877

[65] Ross EA Coughlan RE Flores-Langarica A Lax S Nicholson J Desanti GE. Thymic Function is Maintained During Salmonella-induced Atrophy and Recovery. J Immunol (2012) 189(9):4266–74. 10.4049/jimmunol.1200070 PMC3912538 22993205

[66] Majumdar S Mishra V Nandi S Abdullah M Barman A Raghavan A. Absence of Receptor Guanylyl Cyclase C Enhances Ileal Damage and Reduces Cytokine and Antimicrobial Peptide Production During Oral Salmonella Enterica Serovar Typhimurium Infection. Infect Immun (2018) 86(5):e00799–17. 10.1128/iai.00799-17 29463616 PMC5913844

[67] Sempowski GD Rhein ME Scearce RM Haynes BF. Leukemia Inhibitory Factor is a Mediator of Escherichia Coli Lipopolysaccharide-Induced Acute Thymic Atrophy. Eur J Immunol (2002) 32(11):3066–70. 10.1002/1521-4141(200211)32:11<3066::Aid-immu3066>3.0.Co;2-j 12385026

[68] Norimatsu M Ono T Aoki A Ohishi K Tamura Y. In-Vivo Induction of Apoptosis in Murine Lymphocytes by Bacterial Lipopolysaccharides. J Med Microbiol (1995) 43(4):251–7. 10.1099/00222615-43-4-251 7562985

[69] Chisari FV Northrup RS. Pathophysiologic Effects of Lethal and Immunoregulatory Doses of Cholera Enterotoxin in the Mouse. J Immunol (1974) 113(3):740–9. 4606891

[70] Hussain A Himeno K Mayumi H Kawamura I Tsuru S Nomoto K. Immunomodulatory Effects of Cholera Toxin in Mice. Nat Immun Cell Growth Regul (1989) 8(4):231–44. 2797014

[71] Tsuji T Asano Y Handa T Honma Y Ichinose Y Yokochi T. Induction of Apoptosis in Lymphoid Tissues of Mice After Intramuscular Injection of Enterotoxigenic Escherichia Coli Enterotoxin. Immunobiology (2000) 201(3-4):377–90. 10.1016/s0171-2985(00)80092-3 10776794

[72] Kuchler L Sha LK Giegerich AK Knape T Angioni C Ferreiros N. Elevated Intrathymic sphingosine-1-phosphate Promotes Thymus Involution During Sepsis. Mol Immunol (2017) 90:255–63. 10.1016/j.molimm.2017.08.011 28846923

[73] Kong Y Li Y Zhang W Yuan S Winkler R Krohnert U. Sepsis-Induced Thymic Atrophy Is Associated With Defects in Early Lymphopoiesis. Stem Cells (2016) 34(12):2902–15. 10.1002/stem.2464 27422171

[74] Wang S Lyu C Duan G Meng F Yang Y Yu Y. Streptococcus Suis Serotype 2 Infection Causes Host Immunomodulation Through Induction of Thymic Atrophy. Infect Immun (2020) 88(4):e00950–19. 10.1128/iai.00950-19 31932328 PMC7093123

[75] Starikova EA Golovin AS Vasilyev KA Karaseva AB Serebriakova MK Sokolov AV. Role of Arginine Deiminase in Thymic Atrophy During Experimental Streptococcus Pyogenes Infection. Scand J Immunol (2019) 89(2):e12734. 10.1111/sji.12734 30471128

[76] Chen L-y Ming J Shui-ping L Xue-jun Y. Apoptosis of Mouse Thymocytes and Its Gene Regulation Induced by Listeria Monocytogenesis. Shanghai J Immunol (2001) 06):327–329+333.

[77] Lin YS Huang YT Chen PS Lin CF Jan MS Lei HY. Requirement of I-E Molecule for Thymocyte Apoptosis Induced by Staphylococcal Enterotoxin B In Vivo. Cell Immunol (1999) 193(1):71–9. 10.1006/cimm.1998.1442 10202114

[78] Chen W Kuolee R Austin JW Shen H Che Y Conlan JW. Low Dose Aerosol Infection of Mice With Virulent Type A Francisella Tularensis Induces Severe Thymus Atrophy and CD4+CD8+ Thymocyte Depletion. Microb Pathog (2005) 39(5-6):189–96. 10.1016/j.micpath.2005.08.005 PMC1564440 16257504

[79] Lin YS Chen KH Kuo CF Huang KJ Wu JJ. Induction of Thymocyte Apoptosis in Mice by Yersinia Enterocolitica Products. J Med Microbiol (1998) 47(5):447–54. 10.1099/00222615-47-5-447 9879946

[80] Abramov VM Kosarev IV Motin VL Khlebnikov VS Vasilenko RN Sakulin VK. Binding of LcrV Protein From Yersinia Pestis to Human T-cells Induces Apoptosis, Which is Completely Blocked by Specific Antibodies. Int J Biol Macromol (2019) 122:1062–70. 10.1016/j.ijbiomac.2018.09.054 30218736

[81] Borges M Barreira-Silva P Florido M Jordan MB Correia-Neves M Appelberg R. Molecular and Cellular Mechanisms of Mycobacterium Avium-Induced Thymic Atrophy. J Immunol (2012) 189(7):3600–8. 10.4049/jimmunol.1201525 PMC3593108 22922815

[82] Bottasso O Bay ML Besedovsky H del Rey A. The Immuno-Endocrine Component in the Pathogenesis of Tuberculosis. Scand J Immunol (2007) 66(2-3):166–75. 10.1111/j.1365-3083.2007.01962.x 17635794

[83] Ozeki Y Kaneda K Fujiwara N Morimoto M Oka S Yano I. In Vivo Induction of Apoptosis in the Thymus by Administration of Mycobacterial Cord Factor (Trehalose 6,6’-Dimycolate). Infect Immun (1997) 65(5):1793–9. 10.1128/IAI.65.5.1793-1799.1997 PMC175219 9125563

[84] Wang SD Huang KJ Lin YS Lei HY. Sepsis-Induced Apoptosis of the Thymocytes in Mice. J Immunol (1994) 152(10):5014–21. 8176219

[85] Perez AR Berbert LR Lepletier A Revelli S Bottasso O Silva-Barbosa SD. TNF-Alpha is Involved in the Abnormal Thymocyte Migration During Experimental Trypanosoma Cruzi Infection and Favors the Export of Immature Cells. PloS One (2012) 7(3):e34360. 10.1371/journal.pone.0034360 22461911 PMC3312912

[86] de Meis J Morrot A Farias-de-Oliveira DA Villa-Verde DM Savino W. Differential Regional Immune Response in Chagas Disease. PloS Negl Trop Dis (2009) 3(7):e417. 10.1371/journal.pntd.0000417 19582140 PMC2700264

[87] Roggero E Del Rey A Wildmann J Besedovsky H. Glucocorticoids and Sympathetic Neurotransmitters Modulate the Acute Immune Response to Trypanosoma Cruzi. Ann N Y Acad Sci (2019) 1437(1):83–93. 10.1111/nyas.13946 30088661

[88] Carbajosa S Gea S Chillón-Marinas C Poveda C Del Carmen Maza M Fresno M. Altered Bone Marrow Lymphopoiesis and interleukin-6-dependent Inhibition of Thymocyte Differentiation Contribute to Thymic Atrophy During Trypanosoma Cruzi Infection. Oncotarget (2017) 8(11):17551–61. 10.18632/oncotarget.14886 PMC5392268 28147332

[89] Losada-Barragan M Umana-Perez A Duraes J Cuervo-Escobar S Rodriguez-Vega A Ribeiro-Gomes FL. Thymic Microenvironment is Modified by Malnutrition and Leishmania Infantum Infection. Front Cell Infect Microbiol (2019) 9:252. 10.3389/fcimb.2019.00252 31355153 PMC6639785

[90] Losada-Barragan M Umana-Perez A Cuervo-Escobar S Berbert LR Porrozzi R Morgado FN. Protein Malnutrition Promotes Dysregulation of Molecules Involved in T Cell Migration in the Thymus of Mice Infected With Leishmania Infantum. Sci Rep (2017) 7:45991. 10.1038/srep45991 28397794 PMC5387407

[91] Andrade CF Gameiro J Nagib PR Carvalho BO Talaisys RL Costa FT. Thymic Alterations in Plasmodium Berghei-Infected Mice. Cell Immunol (2008) 253(1-2):1–4. 10.1016/j.cellimm.2008.06.001 18635160

[92] Francelin C Paulino LC Gameiro J Verinaud L. Effects of Plasmodium Berghei on Thymus: High Levels of Apoptosis and Premature Egress of CD4(+)CD8(+) Thymocytes in Experimentally Infected Mice. Immunobiology (2011) 216(10):1148–54. 10.1016/j.imbio.2011.03.009 21601941

[93] Gameiro J Nagib PR Andrade CF Villa-Verde DM Silva-Barbosa SD Savino W. Changes in Cell Migration-Related Molecules Expressed by Thymic Microenvironment During Experimental Plasmodium Berghei Infection: Consequences on Thymocyte Development. Immunology (2010) 129(2):248–56. 10.1111/j.1365-2567.2009.03177.x PMC2814466 19824923

[94] Seixas E Ostler D. Plasmodium Chabaudi Chabaudi (as): Differential Cellular Responses to Infection in Resistant and Susceptible Mice. Exp Parasitol (2005) 110(4):394–405. 10.1016/j.exppara.2005.03.024 15953500

[95] Khanam S Sharma S Pathak S. Lethal and Nonlethal Murine Malarial Infections Differentially Affect Apoptosis, Proliferation, and CD8 Expression on Thymic T Cells. Parasite Immunol (2015) 37(7):349–61. 10.1111/pim.12197 25886201

[96] Chen AL Sun X Wang W Liu JF Zeng X Qiu JF. Activation of the Hypothalamic-Pituitary-Adrenal (HPA) Axis Contributes to the Immunosuppression of Mice Infected With Angiostrongylus Cantonensis. J Neuroinflamm (2016) 13(1):266. 10.1186/s12974-016-0743-z PMC5062856 27733201

[97] Liu Z Su DM Yu ZL Wu F Liu RF Luo SQ. Soluble Antigens From the Neurotropic Pathogen Angiostrongylus Cantonensis Directly Induce Thymus Atrophy in a Mouse Model. Oncotarget (2017) 8(30):48575–90. 10.18632/oncotarget.17836 PMC5564709 28548945

[98] Wellhausen SR Boros DL. Atrophy of the Thymic Cortex in Mice With Granulomatous Schistosomiasis Mansoni. Infect Immun (1982) 35(3):1063–9. 10.1128/iai.35.3.1063-1069.1982 PMC351155 6978289

[99] Li N Ji PY Song LG Lei JX Lv ZY Wu ZD. The Expression of Molecule CD28 and CD38 on CD4⁺/CD8⁺ T Lymphocytes in Thymus and Spleen Elicited by Schistosoma Japonicum Infection in Mice Model. Parasitol Res (2015) 114(8):3047–58. 10.1007/s00436-015-4507-y 26002824

[100] Kawabata M Hosaka Y Kumada M Matsui N Kobayakawa T. Thymocytotoxic Autoantibodies Found in Mice Infected With Schistosoma Japonicum. Infect Immun (1981) 32(2):438–42. 10.1128/iai.32.2.438-442.1981 PMC351462 6972913

[101] Brito VN Souto PC Cruz-Höfling MA Ricci LC Verinaud L. Thymus Invasion and Atrophy Induced by Paracoccidioides Brasiliensis in BALB/c Mice. Med Mycol (2003) 41(2):83–7. 10.1080/mmy.41.2.83.87 12964839

[102] Di Gangi R Alves da Costa T Thomé R Peron G Burger E Verinaud L. Paracoccidioides Brasiliensis Infection Promotes Thymic Disarrangement and Premature Egress of Mature Lymphocytes Expressing Prohibitive Tcrs. BMC Infect Dis (2016) 16:209. 10.1186/s12879-016-1561-8 27189089 PMC4869377

[103] Alves da Costa T Di Gangi R Thomé R Barreto Felisbino M Pires Bonfanti A Lumi Watanabe Ishikawa L. Severe Changes in Thymic Microenvironment in a Chronic Experimental Model of Paracoccidioidomycosis. PloS One (2016) 11(10):e0164745. 10.1371/journal.pone.0164745 27736987 PMC5063316

[104] Sutton P Newcombe NR Waring P Müllbacher A. In Vivo Immunosuppressive Activity of Gliotoxin, a Metabolite Produced by Human Pathogenic Fungi. Infect Immun (1994) 62(4):1192–8. 10.1128/IAI.62.4.1192-1198.1994 PMC186256 7510665

[105] Islam Z Nagase M Yoshizawa T Yamauchi K Sakato N. T-2 Toxin Induces Thymic Apoptosis In Vivo in Mice. Toxicol Appl Pharmacol (1998) 148(2):205–14. 10.1006/taap.1997.8338 9473527

[106] Alvarez F Villena A Zapata A Razquin B. Histopathology of the Thymus in Saprolegnia-infected Wild Brown Trout, Salmo Trutta L. Vet Immunol Immunopathol (1995) 47(1-2):163–72. 10.1016/0165-2427(94)05384-5 8533294

[107] Lv QZ Li DD Han H Yang YH Duan JL Ma HH. Priming With FLO8-deficient Candida Albicans Induces Th1-biased Protective Immunity Against Lethal Polymicrobial Sepsis. Cell Mol Immunol (2020) 5:1–14. 10.1038/s41423-020-00576-6 PMC7642578 33154574

[108] Li Y Wang G Liu Y Tu Y He Y Wang Z. Identification of Apoptotic Cells in the Thymus of Piglets Infected With Highly Pathogenic Porcine Reproductive and Respiratory Syndrome Virus. Virus Res (2014) 189:29–33. 10.1016/j.virusres.2014.04.011 24787009

[109] Stadejek T Stankevicius A Murtaugh MP Oleksiewicz MB. Molecular Evolution of PRRSV in Europe: Current State of Play. Vet Microbiol (2013) 165(1-2):21–8. 10.1016/j.vetmic.2013.02.029. Cited in: Pubmed 23528651

[110] Karniychuk UU Geldhof M Vanhee M Van Doorsselaere J Saveleva TA Nauwynck HJ. Pathogenesis and Antigenic Characterization of a New East European Subtype 3 Porcine Reproductive and Respiratory Syndrome Virus Isolate. BMC Vet Res (2010) 6:30. 10.1186/1746-6148-6-30 20525333 PMC2898778

[111] Feng WH Tompkins MB Xu JS Brown TT Laster SM Zhang HX. Thymocyte and Peripheral Blood T Lymphocyte Subpopulation Changes in Piglets Following In Utero Infection With Porcine Reproductive and Respiratory Syndrome Virus. Virology (2002) 302(2):363–72. 10.1006/viro.2002.1650 12441080

[112] Wang G He Y Tu Y Liu Y Zhou EM Han Z. Comparative Analysis of Apoptotic Changes in Peripheral Immune Organs and Lungs Following Experimental Infection of Piglets With Highly Pathogenic and Classical Porcine Reproductive and Respiratory Syndrome Virus. Virol J (2014) 11:2. 10.1186/1743-422x-11-2 24393149 PMC3892014

[113] Wang G Yu Y Tu Y Li Y Tong J Zhang C. Characterizing the Thymic Lesions in Piglets Infected With Attenuated Strains of Highly Pathogenic Porcine Reproductive and Respiratory Syndrome Virus. Vet Immunol Immunopathol (2015) 168(3-4):258–61. 10.1016/j.vetimm.2015.10.007 26564577

[114] Ogno G Rodriguez-Gomez IM Canelli E Ruedas-Torres I Alvarez B Dominguez J. Impact of PRRSV Strains of Different In Vivo Virulence on the Macrophage Population of the Thymus. Vet Microbiol (2019) 232:137–45. 10.1016/j.vetmic.2019.04.016 31030838

[115] Butler JE Sinkora M Wang G Stepanova K Li Y Cai X. Perturbation of Thymocyte Development Underlies the PRRS Pandemic: A Testable Hypothesis. Front Immunol (2019) 10:1077. 10.3389/fimmu.2019.01077 31156633 PMC6529568

[116] Kolte L Rosenfeldt V Vang L Jeppesen D Karlsson I Ryder LP. Reduced Thymic Size But No Evidence of Impaired Thymic Function in Uninfected Children Born to Human Immunodeficiency Virus-Infected Mothers. Pediatr Infect Dis J (2011) 30(4):325–30. 10.1097/INF.0b013e3182019bc3 21085050

[117] Gimeno IM Witter RL Cortes AL Reed WM. Replication Ability of Three Highly Protective Marek’s Disease Vaccines: Implications in Lymphoid Organ Atrophy and Protection. Avian Pathol (2011) 40(6):573–9. 10.1080/03079457.2011.617725 22107091

[118] Lee LF Heidari M Zhang H Lupiani B Reddy SM Fadly A. Cell Culture Attenuation Eliminates rMd5DeltaMeq-induced Bursal and Thymic Atrophy and Renders the Mutant Virus as an Effective and Safe Vaccine Against Marek’s Disease. Vaccine (2012) 30(34):5151–8. 10.1016/j.vaccine.2012.05.043 22687760

[119] Lee LF Kreager K Heidari M Zhang H Lupiani B Reddy SM. Properties of a Meq-Deleted Rmd5 Marek’s Disease Vaccine: Protection Against Virulent MDV Challenge and Induction of Lymphoid Organ Atrophy are Simultaneously Attenuated by Serial Passage In Vitro. Avian Dis (2013) 57(2 Suppl):491–7. 10.1637/10388-092612-Reg.1 23901766

[120] Yilmaz H Turan N Ozgur NY Helps CR Akay. Detection of Chicken Anemia Virus DNA in the Thymus of Naturally Infected Chicks in Turkey. Avian Dis (2001) 45(2):529–33. 10.2307/1593000 11417840

[121] Kizaki H Suzuki K Tadakuma T Ishimura Y. Adenosine Receptor-Mediated Accumulation of Cyclic AMP-induced T-Lymphocyte Death Through Internucleosomal DNA Cleavage. J Biol Chem (1990) 265(9):5280–4. 10.1016/S0021-9258(19)34118-3 1690738

[122] Nashar TO Williams NA Hirst TR. Cross-Linking of Cell Surface Ganglioside GM1 Induces the Selective Apoptosis of Mature CD8+ T Lymphocytes. Int Immunol (1996) 8(5):731–6. 10.1093/intimm/8.5.731 8671661

[123] Mocchegiani E Santarelli L Costarelli L Cipriano C Muti E Giacconi R. Plasticity of Neuroendocrine-Thymus Interactions During Ontogeny and Ageing: Role of Zinc and Arginine. Ageing Res Rev (2006) 5(3):281–309. 10.1016/j.arr.2006.06.001 16904953

[124] Chantranupong L Scaria SM Saxton RA Gygi MP Shen K Wyant GA. The CASTOR Proteins Are Arginine Sensors for the mTORC1 Pathway. Cell (2016) 165(1):153–64. 10.1016/j.cell.2016.02.035 PMC4808398 26972053

[125] Keating R McGargill MA. Mtor Regulation of Lymphoid Cells in Immunity to Pathogens. Front Immunol (2016) 7:180. 10.3389/fimmu.2016.00180 27242787 PMC4862984

[126] Das P Lahiri A Lahiri A Chakravortty D. Modulation of the Arginase Pathway in the Context of Microbial Pathogenesis: A Metabolic Enzyme Moonlighting as an Immune Modulator. PloS Pathog (2010) 6(6):e1000899. 10.1371/journal.ppat.1000899 20585552 PMC2887468

[127] Napolitano LA Lo JC Gotway MB Mulligan K Barbour JD Schmidt D. Increased Thymic Mass and Circulating Naive CD4 T Cells in HIV-1-infected Adults Treated With Growth Hormone. Aids (2002) 16(8):1103–11. 10.1097/00002030-200205240-00003 12004268

[128] Wang D Muller N McPherson KG Reichardt HM. Glucocorticoids Engage Different Signal Transduction Pathways to Induce Apoptosis in Thymocytes and Mature T Cells. J Immunol (2006) 176(3):1695–702. 10.4049/jimmunol.176.3.1695 16424199

[129] El-Shaikh KA Gabry MS Othman GA. Recovery of Age-Dependent Immunological Deterioration in Old Mice by Thyroxine Treatment. J Anim Physiol Anim Nutr (Berl) (2006) 90(5-6):244–54. 10.1111/j.1439-0396.2005.00602.x 16684146

[130] Hareramadas B Rai U. Cellular Mechanism of Estrogen-Induced Thymic Involution in Wall Lizard: Caspase-Dependent Action. J Exp Zool A Comp Exp Biol (2006) 305(5):396–409. 10.1002/jez.a.260 16526045

[131] De Mello-Coelho V Savino W Postel-Vinay MC Dardenne M. Role of Prolactin and Growth Hormone on Thymus Physiology. Dev Immunol (1998) 6(3-4):317–23. 10.1155/1998/89782. Cited in: Pubmed PMC2276021 9814605

[132] Merrick JC Edelson BT Bhardwaj V Swanson PE Unanue ER. Lymphocyte Apoptosis During Early Phase of Listeria Infection in Mice. Am J Pathol (1997) 151(3):785–92. PMC1857845 9284827

[133] Ito M Nishiyama K Hyodo S Shigeta S Ito T. Weight Reduction of Thymus and Depletion of Lymphocytes of T-dependent Areas in Peripheral Lymphoid Tissues of Mice Infected With Francisella Tularensis. Infect Immun (1985) 49(3):812–8. 10.1128/IAI.49.3.812-818.1985 PMC261284 3875562

[134] Nguyen T Waseem M. Chagas Disease (American Trypanosomiasis). Treasure Island (FL: StatPearls (2020). StatPearls Publishing Copyright © 2020, StatPearls Publishing LLC.

[135] Colato RP Brazão V do Vale GT Santello FH Sampaio PA Tirapelli CR. Cytokine Modulation, Oxidative Stress and Thymic Dysfunctions: Role of Age-Related Changes in the Experimental Trypanosoma Cruzi Infection: Age-related Thymic Dysfunctions and Trypanosoma Cruzi Infection. Cytokine (2018) 111:88–96. 10.1016/j.cyto.2018.08.004 30130728

[136] Beltran-Hortelano I Alcolea V Font M Pérez-Silanes S. The Role of Imidazole and Benzimidazole Heterocycles in Chagas Disease: A Review. Eur J Med Chem (2020) 206:112692. 10.1016/j.ejmech.2020.112692 32818869

[137] Perez AR Morrot A Carvalho VF de Meis J Savino W. Role of Hormonal Circuitry Upon T Cell Development in Chagas Disease: Possible Implications on T Cell Dysfunctions. Front Endocrinol (Lausanne) (2018) 9:334. 10.3389/fendo.2018.00334 29963015 PMC6010535

[138] Morrot A Terra-Granado E Perez AR Silva-Barbosa SD Milicevic NM Farias-de-Oliveira DA. Chagasic Thymic Atrophy Does Not Affect Negative Selection But Results in the Export of Activated CD4+CD8+ T Cells in Severe Forms of Human Disease. PloS Negl Trop Dis (2011) 5(8):e1268. 10.1371/journal.pntd.0001268 21858238 PMC3156684

[139] Matloubian M Lo CG Cinamon G Lesneski MJ Xu Y Brinkmann V. Lymphocyte Egress From Thymus and Peripheral Lymphoid Organs is Dependent on S1P Receptor 1. Nature (2004) 427(6972):355–60. 10.1038/nature02284 14737169

[140] Lepletier A de Almeida L Santos L da Silva Sampaio L Paredes B Gonzalez FB. Early Double-Negative Thymocyte Export in Trypanosoma Cruzi Infection is Restricted by Sphingosine Receptors and Associated With Human Chagas Disease. PloS Negl Trop Dis (2014) 8(10):e3203. 10.1371/journal.pntd.0003203 25330249 PMC4199546

[141] Nardy AF Luiz da Silva Filho J Perez AR de Meis J Farias-de-Oliveira DA Penha L. Trans-Sialidase From Trypanosoma Cruzi Enhances the Adhesion Properties and Fibronectin-Driven Migration of Thymocytes. Microbes Infect (2013) 15(5):365–74. 10.1016/j.micinf.2013.02.003 23481510

[142] Farias-de-Oliveira DA Cotta-de-Almeida V Villa-Verde DM Riederer I Meis J Savino W. Fibronectin Modulates Thymocyte-Thymic Epithelial Cell Interactions Following Trypanosoma Cruzi Infection. Mem Inst Oswaldo Cruz (2013) 108(7):825–31. 10.1590/0074-0276130071 PMC3970635 24271041

[143] Silva-Monteiro E Reis Lorenzato L Kenji Nihei O Junqueira M Rabinovich GA Hsu DK. Altered Expression of Galectin-3 Induces Cortical Thymocyte Depletion and Premature Exit of Immature Thymocytes During Trypanosoma Cruzi Infection. Am J Pathol (2007) 170(2):546–56. 10.2353/ajpath.2007.060389 PMC1851869 17255323

[144] Gonzalez FB Calmon-Hamaty F No Seara Cordeiro S Fernandez Bussy R Spinelli SV D’Attilio L. Trypanosoma Cruzi Experimental Infection Impacts on the Thymic Regulatory T Cell Compartment. PloS Negl Trop Dis (2016) 10(1):e0004285. 10.1371/journal.pntd.0004285 26745276 PMC4706328

[145] Gonzalez FB Villar SR Fernandez Bussy R Martin GH Perol L Manarin R. Immunoendocrine Dysbalance During Uncontrolled T. Cruzi Infection is Associated With the Acquisition of a Th-1-like Phenotype by Foxp3(+) T Cells. Brain Behav Immun (2015) 45:219–32. 10.1016/j.bbi.2014.11.016 PMC7126853 25483139

[146] Besedovsky HO del Rey A. Immune-Neuro-Endocrine Interactions: Facts and Hypotheses. Endocr Rev (1996) 17(1):64–102. 10.1210/edrv-17-1-64 8641224

[147] Farias-de-Oliveira DA Villa-Verde DM Nunes Panzenhagen PH Silva dos Santos D Berbert LR Savino W. Caspase-8 and Caspase-9 Mediate Thymocyte Apoptosis in Trypanosoma Cruzi Acutely Infected Mice. J Leukoc Biol (2013) 93(2):227–34. 10.1189/jlb.1211589 23159925

[148] Bonney KM Engman DM. Autoimmune Pathogenesis of Chagas Heart Disease: Looking Back, Looking Ahead. Am J Pathol (2015) 185(6):1537–47. 10.1016/j.ajpath.2014.12.023 PMC4450315 25857229

[149] Perez AR Roggero E Nicora A Palazzi J Besedovsky HO Del Rey A. Thymus Atrophy During Trypanosoma Cruzi Infection is Caused by an Immuno-Endocrine Imbalance. Brain Behav Immun (2007) 21(7):890–900. 10.1016/j.bbi.2007.02.004 17412557

[150] Roggero E Perez AR Tamae-Kakazu M Piazzon I Nepomnaschy I Besedovsky HO. Endogenous Glucocorticoids Cause Thymus Atrophy But are Protective During Acute Trypanosoma Cruzi Infection. J Endocrinol (2006) 190(2):495–503. 10.1677/joe.1.06642 16899582

[151] Krishnan N Thellin O Buckley DJ Horseman ND Buckley AR. Prolactin Suppresses Glucocorticoid-Induced Thymocyte Apoptosis In Vivo. Endocrinology (2003) 144(5):2102–10. 10.1210/en.2003-0053 12697719

[152] Biswas R Roy T Chattopadhyay U. Prolactin Induced Reversal of Glucocorticoid Mediated Apoptosis of Immature Cortical Thymocytes is Abrogated by Induction of Tumor. J Neuroimmunol (2006) 171(1-2):120–34. 10.1016/j.jneuroim.2005.09.014 16289331

[153] Savino W. Endocrine Immunology of Chagas Disease. Front Horm Res (2017) 48:160–75. 10.1159/000452914 28245460

[154] Lepletier A de Carvalho VF Rodrigues e Silva PM Villar S Perez AR Savino W. Trypanosoma Cruzi Disrupts Thymic Homeostasis by Altering Intrathymic and Systemic Stress-Related Endocrine Circuitries. PloS Negl Trop Dis (2013) 7(11):e2470. 10.1371/journal.pntd.0002470 24324845 PMC3852165

[155] Loria RM. Antiglucocorticoid Function of Androstenetriol. Psychoneuroendocrinology (1997) 22 Suppl 1:S103–8. 10.1016/s0306-4530(97)00005-x 9264155

[156] Hampl R Vondra K. [Natural Antiglucocorticoids]. Vnitr Lek (2006) 52(10):973–8. 17063813

[157] Chen Y Qiao S Tuckermann J Okret S Jondal M. Thymus-Derived Glucocorticoids Mediate Androgen Effects on Thymocyte Homeostasis. FASEB J (2010) 24(12):5043–51. 10.1096/fj.10-168724 PMC2992366 20798244

[158] Mucci J Mocetti E Leguizamon MS Campetella O. A Sexual Dimorphism in Intrathymic Sialylation Survey is Revealed by the Trans-Sialidase From Trypanosoma Cruzi. J Immunol (2005) 174(8):4545–50. 10.4049/jimmunol.174.8.4545 15814675

[159] Filipin Mdel V Caetano LC Brazao V Santello FH Toldo MP do Prado JC Jr. DHEA and Testosterone Therapies in Trypanosoma Cruzi-Infected Rats are Associated With Thymic Changes. Res Vet Sci (2010) 89(1):98–103. 10.1016/j.rvsc.2010.01.016 20202657

[160] Engdahl C Lagerquist MK Stubelius A Andersson A Studer E Ohlsson C. Role of Androgen and Estrogen Receptors for the Action of Dehydroepiandrosterone (DHEA). Endocrinology (2014) 155(3):889–96. 10.1210/en.2013-1561 24424045

[161] Linhares-Lacerda L Palu CC Ribeiro-Alves M Paredes BD Morrot A Garcia-Silva MR. Differential Expression of microRNAs in Thymic Epithelial Cells From Trypanosoma Cruzi Acutely Infected Mice: Putative Role in Thymic Atrophy. Front Immunol (2015) 6:428. 10.3389/fimmu.2015.00428 26347748 PMC4543887

[162] Acuna SM Floeter-Winter LM Muxel SM. Micrornas: Biological Regulators in Pathogen-Host Interactions. Cells (2020) 9(1):113. 10.3390/cells9010113. Cited in: Pubmed PMC7016591 31906500

[163] Lévêque MF Lachaud L Simon L Battery E Marty P Pomares C. Place of Serology in the Diagnosis of Zoonotic Leishmaniases With a Focus on Visceral Leishmaniasis Due to Leishmania Infantum. Front Cell Infect Microbiol (2020) 10:67. 10.3389/fcimb.2020.00067 32158704 PMC7052174

[164] de Koning-Ward TF Dixon MW Tilley L Gilson PR. Plasmodium Species: Master Renovators of Their Host Cells. Nat Rev Microbiol (2016) 14(8):494–507. 10.1038/nrmicro.2016.79 27374802

[165] Ashley EA Pyae Phyo A Woodrow CJ. Malaria. Lancet (2018) 391(10130):1608–21. 10.1016/s0140-6736(18)30324-6 29631781

[166] Stephens R Culleton RL Lamb TJ. The Contribution of Plasmodium Chabaudi to Our Understanding of Malaria. Trends Parasitol (2012) 28(2):73–82. 10.1016/j.pt.2011.10.006 22100995 PMC4040349

[167] Carvalho LJ Ferreira-da-cruz MF Daniel-Ribeiro CT Pelajo-Machado M Lenzi HL. Plasmodium Berghei ANKA Infection Induces Thymocyte Apoptosis and Thymocyte Depletion in CBA Mice. Mem Inst Oswaldo Cruz (2006) 101(5):523–8. 10.1590/s0074-02762006000500007 17072456

[168] Pinna RA Silva-Dos-Santos D Perce-da-Silva DS Oliveira-Ferreira J Villa-Verde DM De Luca PM. Malaria-Cutaneous Leishmaniasis Co-Infection: Influence on Disease Outcomes and Immune Response. Front Microbiol (2016) 7:982. 10.3389/fmicb.2016.00982 27446022 PMC4921482

[169] Wang QP Lai DH Zhu XQ Chen XG Lun ZR. Human Angiostrongyliasis. Lancet Infect Dis (2008) Oct 8(10):621–30. 10.1016/s1473-3099(08)70229-9 18922484

[170] Sohal RJ Gilotra TS Lui F. Angiostrongylus Cantonensis (Angiostrongliasis). (2020) StatPearls. Treasure Island (FL): StatPearls Publishing. 32310527

[171] Schwartz C Fallon PG. Schistosoma “Eggs-Iting” the Host: Granuloma Formation and Egg Excretion. Front Immunol (2018) 9:2492. 10.3389/fimmu.2018.02492 30459767 PMC6232930

[172] McManus DP Dunne DW Sacko M Utzinger J Vennervald BJ Zhou XN. Schistosomiasis. Nat Rev Dis Primers (2018) 4(1):13. 10.1038/s41572-018-0013-8 30093684

[173] Gryseels B. Schistosomiasis. Infect Dis Clin North Am (2012) 26(2):383–97. 10.1016/j.idc.2012.03.004 22632645

[174] Zhang J Wang Y Aili A Sun X Pang X Ge Q. Aire-Th1 Biased Progressive Autoimmunity in Aged Deficient Mice Accelerated Thymic Epithelial Cell Senescence. Aging Dis (2019) 10(3):497–509. 10.14336/ad.2018.0608 31164995 PMC6538216

[175] Majumdar S Adiga V Raghavan A Rananaware SR Nandi D. Comparative Analysis of Thymic Subpopulations During Different Modes of Atrophy Identifies the Reactive Oxygen Species Scavenger, N-acetyl Cysteine, to Increase the Survival of Thymocytes During Infection-Induced and Lipopolysaccharide-Induced Thymic Atrophy. Immunology (2019) 157(1):21–36. 10.1111/imm.13043 30659606 PMC6459778

[176] Schwabe R Luedde T. Apoptosis and Necroptosis in the Liver: A Matter of Life and Death. Nat Rev Gastroenterol Hepatol (2018) 15(12):738–52. 10.1038/s41575-018-0065-y PMC6490680 30250076

[177] Wang K Shi Y Denhardt D. Osteopontin Regulates Hindlimb-Unloading-Induced Lymphoid Organ Atrophy and Weight Loss by Modulating Corticosteroid Production. Proc Natl Acad Sci U States America (2007) 104(37):14777–82. 10.1073/pnas.0703236104 PMC1976226 17785423

[178] Guha I Bhuniya A Nandi P Dasgupta S Sarkar A Saha A. Neem Leaf Glycoprotein Reverses Tumor-Induced and Age-Associated Thymic Involution to Maintain Peripheral CD8 T Cell Pool. Immunotherapy (2020) 12(11):799–818. 10.2217/imt-2019-0168 32698648

[179] Zhang J Li S Li L Li M Guo C Yao J. Exosome and Exosomal microRNA: Trafficking, Sorting, and Function. Genomics Proteomics Bioinf (2015) 13(1):17–24. 10.1016/j.gpb.2015.02.001 PMC4411500 25724326

[180] Coakley G Buck AH Maizels RM. Host Parasite communications-Messages From Helminths for the Immune System: Parasite Communication and Cell-Cell Interactions. Mol Biochem Parasitol (2016) 208(1):33–40. 10.1016/j.molbiopara.2016.06.003 27297184 PMC5008435

[181] Silverman JM Clos J Horakova E Wang AY Wiesgigl M Kelly I. Leishmania Exosomes Modulate Innate and Adaptive Immune Responses Through Effects on Monocytes and Dendritic Cells. J Immunol (2010) 185(9):5011–22. 10.4049/jimmunol.1000541 20881185

[182] Szempruch AJ Sykes SE Kieft R Dennison L Becker AC Gartrell A. Extracellular Vesicles From Trypanosoma Brucei Mediate Virulence Factor Transfer and Cause Host Anemia. Cell (2016) 164(1-2):246–57. 10.1016/j.cell.2015.11.051. Cited in: Pubmed PMC4715261 26771494

[183] Twu O de Miguel N Lustig G Stevens GC Vashisht AA Wohlschlegel JA. Trichomonas Vaginalis Exosomes Deliver Cargo to Host Cells and Mediate Host∶Parasite Interactions. PloS Pathog (2013) 9(7):e1003482. 10.1371/journal.ppat.1003482 23853596 PMC3708881

[184] Geiger A Hirtz C Bécue T Bellard E Centeno D Gargani D. Exocytosis and Protein Secretion in Trypanosoma. BMC Microbiol (2010) Jan 26 10:20. 10.1186/1471-2180-10-20 PMC3224696 20102621

[185] Atyame Nten CM Sommerer N Rofidal V Hirtz C Rossignol M Cuny G. Excreted/Secreted Proteins From Trypanosome Procyclic Strains. J BioMed Biotechnol (2010) 2010:212817. 10.1155/2010/212817 20011064 PMC2789517

[186] Zhu L Liu J Dao J Lu K Li H Gu H. Molecular Characterization of S. Japonicum Exosome-Like Vesicles Reveals Their Regulatory Roles in Parasite-Host Interactions. Sci Rep (2016) 6:25885. 10.1038/srep25885 27172881 PMC4865838

[187] Garcia-Silva MR Cabrera-Cabrera F das Neves RF Souto-Padrón T de Souza W Cayota A. Gene Expression Changes Induced by Trypanosoma Cruzi Shed Microvesicles in Mammalian Host Cells: Relevance of tRNA-derived Halves. BioMed Res Int (2014) 2014:305239. 10.1155/2014/305239 24812611 PMC4000953

[188] Wang L Li Z Shen J Liu Z Liang J Wu X. Exosome-Like Vesicles Derived by Schistosoma Japonicum Adult Worms Mediates M1 Type Immune- Activity of Macrophage. Parasitol Res (2015) 114(5):1865–73. 10.1007/s00436-015-4373-7 25855345

[189] Oliveira DL Freire-de-Lima CG Nosanchuk JD Casadevall A Rodrigues ML Nimrichter L. Extracellular Vesicles From Cryptococcus Neoformans Modulate Macrophage Functions. Infect Immun (2010) 78(4):1601–9. 10.1128/iai.01171-09 PMC2849392 20145096

[190] Buck AH Coakley G Simbari F McSorley HJ Quintana JF Le Bihan T. Exosomes Secreted by Nematode Parasites Transfer Small RNAs to Mammalian Cells and Modulate Innate Immunity. Nat Commun (2014) 5:5488. 10.1038/ncomms6488 25421927 PMC4263141

[191] Majumdar S Nandi D. Thymic Atrophy: Experimental Studies and Therapeutic Interventions. Scand J Immunol (2018) 87(1):4–14. 10.1111/sji.12618 28960415

[192] Müller F Svardal AM Nordoy I Berge RK Aukrust P Frøland SS. Virological and Immunological Effects of Antioxidant Treatment in Patients With HIV Infection. Eur J Clin Invest (2000) 30(10):905–14. 10.1046/j.1365-2362.2000.00727.x 11029606

[193] Liang C Liao J Deng Z Song C Zhang J Zabeau L. Leptin Attenuates Lipopolysaccharide-Induced Apoptosis of Thymocytes Partially Via Down-Regulation of cPLA2 and P38 MAPK Activation. Int Immunopharmacol (2013) 15(3):620–7. 10.1016/j.intimp.2013.01.014 23376443

[194] Brazao V Colato RP Santello FH Vale GTD Gonzaga NA Tirapelli CR. Effects of Melatonin on Thymic and Oxidative Stress Dysfunctions During Trypanosoma Cruzi Infection. J Pineal Res (2018) 65(3):e12510. 10.1111/jpi.12510 29781553

[195] Prattichizzo F Giuliani A Sabbatinelli J Mensà E De Nigris V La Sala L. Extracellular Vesicles Circulating in Young Organisms Promote Healthy Longevity. J Extracell Vesicles (2019) 8(1):1656044. 10.1080/20013078.2019.1656044 31489148 PMC6713086

[196] Wang W Wang L Ruan L Oh J Dong X Zhuge Q. Extracellular Vesicles Extracted From Young Donor Serum Attenuate Inflammaging Via Partially Rejuvenating Aged T-cell Immunotolerance. FASEB J (2018) 21(11):32. 10.1096/fj.201800059R PMC6181631 29782203

[197] Banfai K Garai K Ernszt D Pongracz JE Kvell K. Transgenic Exosomes for Thymus Regeneration. Front Immunol (2019) 10:862. 10.3389/fimmu.2019.00862 31110503 PMC6499203

[198] Leposavić G Ugresić N Pejcić-Karapetrović B Mićić M. Castration of Sexually Immature Rats Affects Sympathetic Innervation of the Adult Thymus. Neuroimmunomodulation (2000) 7(2):59–67. 10.1159/000026421 10686514

[199] Slota C Shi A Chen G Bevans M Weng NP. Norepinephrine Preferentially Modulates Memory CD8 T Cell Function Inducing Inflammatory Cytokine Production and Reducing Proliferation in Response to Activation. Brain Behav Immun (2015) 46:168–79. 10.1016/j.bbi.2015.01.015 PMC4414741 25653192

[200] Kvetnanský R Pacák K Fukuhara K Viskupic E Hiremagalur B Nankova B. Sympathoadrenal System in Stress. Interaction With the Hypothalamic-Pituitary-Adrenocortical System. Ann N Y Acad Sci (1995) 771:131–58. 10.1111/j.1749-6632.1995.tb44676.x 8597393

[201] Leander S Håkanson R Rosell S Folkers K Sundler F Tornqvist K. A Specific Substance P Antagonist Blocks Smooth Muscle Contractions Induced by non-Cholinergic, non-Adrenergic Nerve Stimulation. Nature (1981) 294(5840):467–9. 10.1038/294467a0 6171733

[202] Lucchese A. Streptococcus Mutans Antigen I/II and Autoimmunity in Cardiovascular Diseases. Autoimmun Rev (2017) 16(5):456–60. 10.1016/j.autrev.2017.03.009 28286107

[203] Novotna M Podzimek S Broukal Z Lencova E Duskova J. Periodontal Diseases and Dental Caries in Children With Type 1 Diabetes Mellitus. Mediators Inflammation (2015) 2015:379626. 10.1155/2015/379626 PMC4539482 26347009

